# Studying intrapulmonary pharmacokinetics for tuberculosis treatment: a systematic review of methodology

**DOI:** 10.1093/jac/dkaf274

**Published:** 2025-08-14

**Authors:** Isabella van der Feltz, Haini Wen, Rob E Aarnoutse, Cecile Magis-Escurra, Elin M Svensson, Lindsey H M te Brake

**Affiliations:** Department of Pharmacy, Pharmacology and Toxicology, Radboud University Medical Center, Nijmegen, The Netherlands; Department of Pharmacy, Uppsala University, Uppsala, Sweden; Department of Pharmacy, Pharmacology and Toxicology, Radboud University Medical Center, Nijmegen, The Netherlands; Department of Pulmonology, Radboud University Medical Center, Nijmegen, The Netherlands; Department of Pharmacy, Pharmacology and Toxicology, Radboud University Medical Center, Nijmegen, The Netherlands; Department of Pharmacy, Uppsala University, Uppsala, Sweden; Department of Pharmacy, Pharmacology and Toxicology, Radboud University Medical Center, Nijmegen, The Netherlands

## Abstract

**Objectives:**

Drug concentrations at the site of disease in pulmonary tuberculosis (TB) remain limitedly available, while adequate exposures of anti-TB drugs in the lungs are required for sterilization of lesions. Intrapulmonary concentration data could benefit TB treatment optimization. We conducted a systematic review to identify methods that can be used for sampling, quantifying, describing and predicting intrapulmonary pharmacokinetics of anti-TB drugs in humans.

**Methods:**

Two systematic search strategies were conducted in databases Embase and PubMed, last searched on 18 July 2024. In total, 253 studies were identified, and their applied methods were classified into three different categories: (i) sampling techniques, (ii) quantitative analysis and (iii) modelling methods. All types of pulmonary diseases were included in the search.

**Results:**

Sputum sampling was reported as sampling method in nine studies, tissue biopsy in 51, bronchoalveolar lavage in 115, bronchoscopic microsampling in eight, bronchoabsorption in one and microdialysis in 12 studies. LC-MS/MS, the gold standard for drug quantification in biological samples, was used in 67 studies. Other quantification methods included positron emission tomography, reported in 12 studies and matrix-assisted laser desorption ionization mass spectrometry on lung tissue in three studies. For prediction and description of (pre)clinical intrapulmonary concentration data, population pharmacokinetic modelling was reported in 32 studies and physiologically based pharmacokinetic modelling in 35 studies.

**Conclusions:**

Many of the identified methods are associated with considerable limitations including invasiveness, complexity, cost and lack of standardization. Most importantly, the method of choice must provide adequate representation of site of disease pharmacokinetics. Determining the best approach for studying intrapulmonary pharmacokinetics involves careful consideration of all these factors.

## Introduction

Tuberculosis (TB) remains the most lethal infectious disease, causing ∼1.3 million deaths in 2022.^1^ It typically manifests as a pulmonary infection, but can also affect other parts of the body. TB infection and disease start with inhalation of droplets carrying *Mycobacterium tuberculosis*, followed by phagocytosis of bacilli by alveolar macrophages in the lungs. A complex inflammatory response ensues, resulting in the formation of hallmark lesions called granulomas, which are characterized by immunological control and bacterial persistence.^2^ Granulomas come in different types and can be categorized on the basis of features such as size, extent of necrosis and abundance of specific immune cells.^3–5^ In more advanced disease stages, liquification of the granuloma centre may lead to destruction of lung parenchyma and vascularization, resulting in cavity formation.^6,7^

Pharmacokinetic-pharmacodynamic (PK-PD) analyses are usually based on anti-TB drug exposures quantified in plasma, although plasma concentrations do not necessarily correlate with intrapulmonary concentrations.^8–10^ As sampling from the lungs is more invasive and complex than plasma sampling, intrapulmonary PK data are scarce. This makes it hard to evaluate whether dosing regimens result in adequate drug exposure and sufficient bactericidal or sterilizing effect at the site of disease.^8^ The limited available data suggest that drug exposure achieved in lungs and pulmonary granuloma is both drug-specific and lesion-specific.^8,11–17^ For instance, one study found that several anti-TB drugs, including rifampicin and pyrazinamide, penetrate into rabbit pulmonary granulomas to a different extent.^8^ Other studies have demonstrated that lesion type and heterogeneity influence drug penetration into granulomas.^11,12^ Moreover, it has been shown that the extent of lesion penetration (and thus drug exposure) affects the sterilizing efficacy against mycobacterial populations there.^11–17^

As suboptimal exposure can lead to the necessity of prolonging treatment or even treatment failure,^9,18^ more studies into site of disease PK are needed as a base for dose optimization of anti-TB drugs and the rational design of regimens.

The relevance of studying *in vivo* site of disease PK of anti-TB drugs for PK-PD analyses and drug development has been addressed elsewhere,^9,19^ but a clear overview of the diverse methods available to study intrapulmonary PK specifically in humans is currently lacking. Therefore, our aim was to conduct a systematic review to identify the available methods for sampling, quantifying, describing and predicting intrapulmonary concentrations in humans. Search strategies were not limited to TB or anti-TB drugs only, as the objective was to identify all available methods that can be applied in humans with TB, also those used in other pulmonary diseases.

## Search and article selection

Two parallel searches on the topic of intrapulmonary drug concentrations were performed in databases Embase and PubMed. Search strategies and inclusion/exclusion criteria can be found in the Supplementary section S1 (available as Supplementary data at *JAC* Online). No terms focusing on TB or specific anti-TB drugs were used.

First, a general search strategy was designed and executed. Articles were screened for eligibility based on title and abstract. Peer-reviewed articles in which intrapulmonary drug concentrations were sampled, quantified, described or predicted in human participants were included. Reviews, conference abstracts and non-English articles were excluded. Articles about *in vitro* and animal studies were also excluded. All eligible articles were combined, and duplicates removed, after which full articles were screened again for eligibility.

Second, a separate search was conducted to identify pulmonary physiologically based pharmacokinetic (PBPK) models. This was necessary, because PBPK models rely on a combination of *in vitro*, animal and human data, which we would miss with the general search strategy. Articles in which a PBPK model was developed or used to predict intrapulmonary drug concentrations in humans were included. The lung part of the model should consist of multiple compartments, but one-compartment lung models were allowed when animal or human tissue/fluid concentration data were collected as well. Articles were combined and duplicates removed, after which full articles were assessed for eligibility. Again, reviews, conference abstracts and non-English articles were excluded.

Results were reported in accordance with the Preferred Reporting Items for Systematic Reviews and Meta-Analyses (PRISMA) 2020 (see Supplementary materials section S3, Tables S6 and S7). Abstract screening and article selection were performed by a single reviewer (I.v.d.F.). No automation tools were used in the process. Information from the selected articles was collected by a single reviewer and gathered into an overview in Microsoft Excel. Endnote 21 was used for managing references.

## Results

The general search strategy was conducted on 18 July 2024. In total, 1920 and 1122 records were identified in Embase and PubMed, respectively. After title and abstract screening, 178 and 51 articles remained, respectively. Subsequently, 30 duplicates were removed. The remaining 199 articles were screened to assess their eligibility and another 35 were removed, leaving 164 articles. Citation searching of the reference lists was performed, leading to another 65 articles that fulfilled the inclusion criteria. The general search thus resulted in a total of 228 articles.

The PBPK search was conducted in Embase and PubMed on 18 July 2024. In total, 514 and 456 records were identified in the respective databases; 347 duplicates were removed, and the remaining 623 articles were screened to assess eligibility. After exclusion of 590 articles and already identified with the general search, there were 25 new PBPK articles. No citation searching was performed.

**Figure 1.**
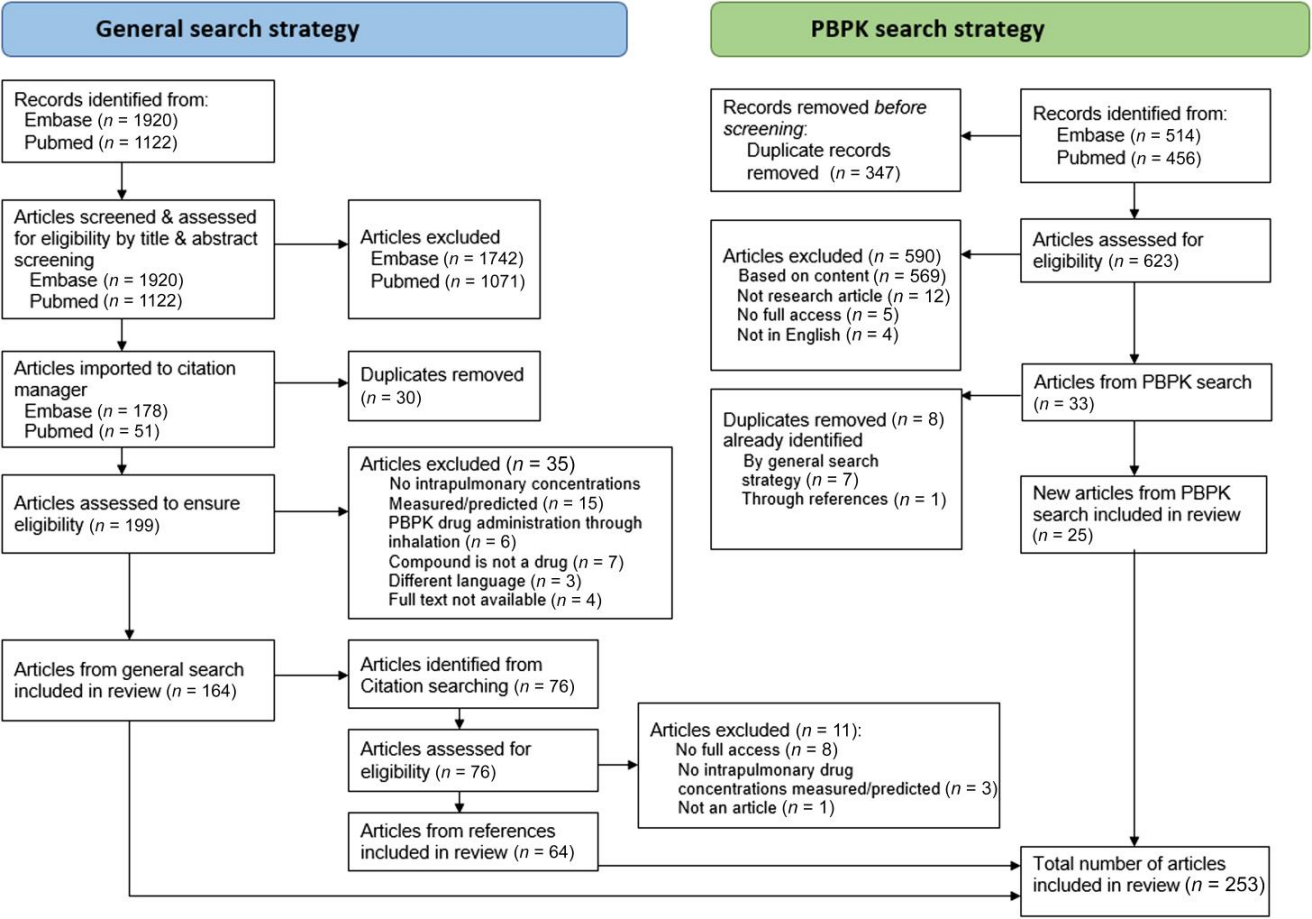
Summary of the article selection process.

The article screening and selection process is shown in Figure 1. Combined, the two search strategies resulted in 253 unique articles. For the full list of included studies from both strategies, including references, see the Supplementary materials section S2.

## Sampling intrapulmonary tissues and fluids

**Figure 2.**
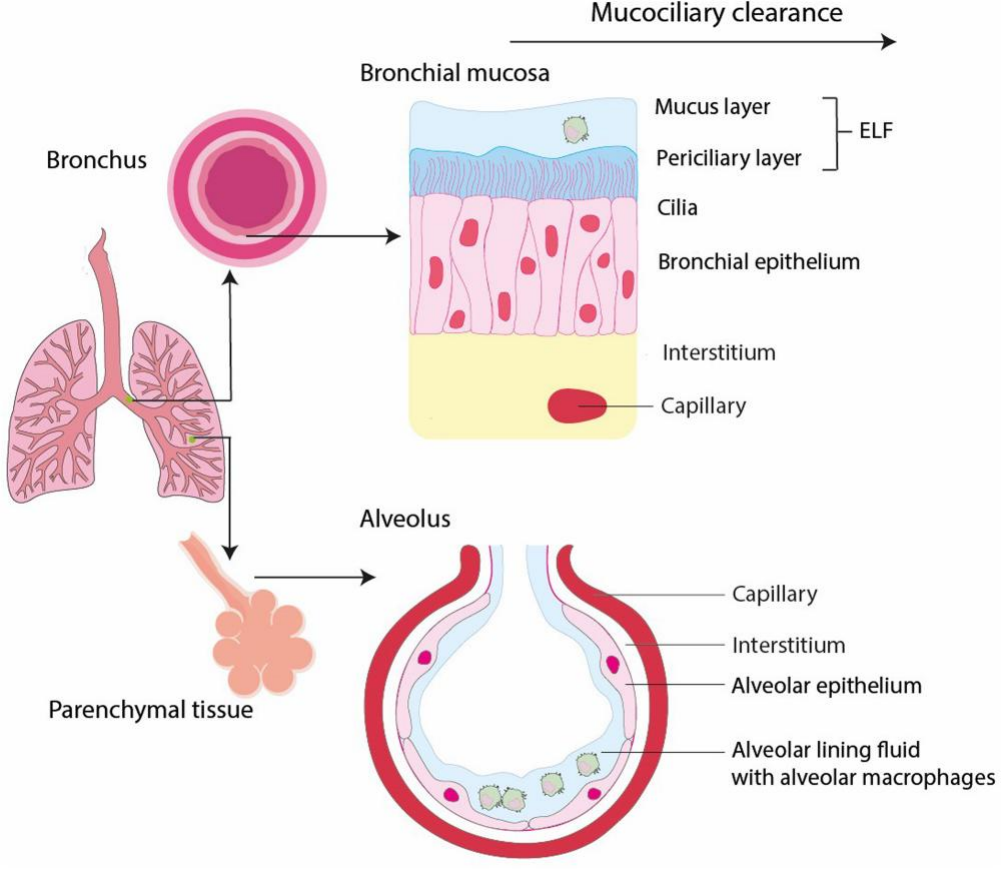
Overview of lung anatomy and physiology. Image created with Adobe Illustrator, partly using images from Servier Medical Art. Servier Medical Art by Servier is licensed under a Creative Commons Attribution 4.0 License (https://creativecommons.org/licenses/by/4.0/).

The lung can anatomically and functionally be divided into different compartments, shown in Figure 2. In the lung parenchyma, alveolar epithelial cells (Figure 2, lower part) are positioned close to the capillaries to facilitate oxygen and carbon dioxide exchange between blood and air.^20^

The bronchial epithelium is covered with cilia and a dynamic aqueous substance, the epithelial lining fluid (ELF)^21,22^ (Figure 2, upper part). Distribution of drug molecules from the capillaries, across the interstitium and into the ELF occurs mainly through passive diffusion.^23^ The ELF contains various types of alveolar cells (ACs), mainly alveolar macrophages (AMs).^24^

In respiratory tract infections, ELF and AMs are considered the extracellular and intracellular sites of infection.^25,26^ In pulmonary TB, granulomas are mainly formed in the lung parenchyma.^27^ The granuloma is the site of disease, with caseum, the necrotic material at the centre of the granuloma, representing the extracellular site, and infected macrophages representing the intracellular sites.^9^

The cilia of the epithelial cells move and propel mucus upwards, thereby providing defence against infections.^28^ Mucus coughed up from the lower airways contains other components (e.g. saliva, cells from the upper airways) and on exit this mixture is called sputum.^21^

Terminology regarding fluid from the airways is not always consistent in literature. In this review, ELF refers to fluid collected from the airways with catheters.

### Sputum

Sputum is collected from patients with respiratory symptoms to determine a responsible pathogen,^29^ normally through expectoration or induction.^29,30^ Expectoration is the act of coughing up sputum, whereas induction also involves the inhalation of nebulized saline to induce fluid production before expectoration.^29,31,32^ Sputum can also be obtained with suction via tracheostoma or bronchoscopy.^33,34^

Sputum is not a homogeneous liquid and sample preparation procedures involve dilution with buffer and centrifugation for homogenization, as observed in the nine articles that measured antibiotic concentrations in sputum samples (see Supplementary section S2; Supplementary Table S1; 1–9). Compared to other methods for obtaining intrapulmonary samples, sputum collection is relatively simple and non-invasive. However, sputum composition varies depending on the patient, infection and disease state, and sputum quality is known to affect microbiological evaluation and diagnosis.^33,35,36^ Whether quality and composition affect the measurement of drug concentrations in sputum samples was not reported in the articles identified, but should be considered as part of the bio-analytical method validation in future studies.

### Tissue biopsy

Two major methods for lung tissue sampling were identified in the included studies: open lung biopsy for sampling parenchymal tissue and endobronchial biopsy for sampling bronchial mucosa tissue. There are also other methods, such as transthoracic needle biopsy and transbronchial (cryo)biopsy,^37–39^ but these were not identified.

Parenchymal tissue biopsies taken during pulmonary surgery are limited to patients already scheduled for this intervention. Bronchial mucosa biopsies are taken during a bronchoscopy, a procedure that involves inserting a flexible tube with a small camera (bronchoscope) into the airways. Tissue biopsy and subsequent analysis of drug concentration in tissue samples were reported in 51 articles (Supplementary section S2; Supplementary Table S1; 8–59). Of these, 25 articles quantified concentrations in parenchymal tissue, 18 in bronchial mucosa tissue, six articles in both, one article in epithelial tissue obtained with a bronchial brush and one article in endobronchial lymph nodes. Sampling tissue from bronchial mucosa is often paired with bronchoalveolar lavage (BAL), as both require a bronchoscope. For MALDI-MS on intact tissue after biopsy, see the ‘MALDI-MS’ section.

After biopsy, blood is removed from the tissue by rinsing with water (which may cause diffusion of drug out of the tissue), and/or drying with sterile paper. Sample preparation involves methods that homogenize the tissue, for example mechanically with beads, pestle or rotor system (11 articles) or by ultrasonication (15 articles). No articles described homogenization solely through enzymatic or chemical dissolution. After homogenization, the supernatant is obtained by centrifugation, and analyte extraction is performed as part of the bio-analysis, which was not detailed in most of the reviewed articles.

With homogenizing procedures, tissue structure is destroyed.^40^ Homogenization needs to be complete to ensure release of all intracellular and interstitial fluids, otherwise there is a risk of underestimation of the drug content. There is also a risk of overestimation of the available active drug content, as only the total concentration in tissue is measured and the degree of protein binding is not considered. An additional disadvantage of homogenization is that all information on drug location is lost. An alternative method, which retains spatial information in tissue, is laser capture microdissection (LCM). This method involves the cutting and isolation of a small tissue section with a laser and extracting drugs from this specific part before analysis with liquid chromatography-tandem mass spectrometry (LC-MS/MS).^41^ Only one article used this method on lymph node tissue of children with TB,^10^ but it has been used frequently in animal studies and could be considered when designing studies obtaining human lung tissue samples.

### ELF sampling

#### Bronchoalveolar lavage (BAL)

A well-known method for sampling ELF is BAL conducted during bronchoscopy. After the administration of local sedation (e.g. lidocaine) to the participant, the bronchoscope is inserted via the nose (or mouth) into the middle lobe of the right lung.^42,43^ A saline solution is instilled and retrieved by suction, and this procedure is repeated several times to obtain multiple aliquots.

BAL was performed and concentrations were measured in BAL fluid in 115 articles (Supplementary section S2; Supplementary Table S1; 8, 36–142). BAL fluid is centrifuged to separate the ELF (supernatant) from the cells (mostly AMs) and both are frozen until analysis. BAL fluid has been diluted and, therefore, original drug concentrations in the ELF are calculated using the urea dilution method first described by Rennard *et al*.^44^ Urea concentrations are measured in BAL fluid and plasma. Urea diffuses quickly, and using the assumption that urea concentrations in ELF and plasma are equal, drug concentrations in BAL fluid are corrected. The cells in pellet suspension can be counted with a hemacytometer and the drug concentration in ACs can be calculated using the mean cellular volume.^45–48^

Drug concentrations measured in BAL fluid are highly variable, and sensitive to technical errors and differences in methodology.^23,49^ During the procedure, diffusion of urea from surrounding tissues and blood into BAL fluid can lead to overestimation of ELF volume and underestimation of drug concentrations.^23,49,50^ Overestimation has also been reported and is possibly caused by extraction of drug from surrounding tissue.^50,51^ In addition to these limitations, BAL is normally performed at only one timepoint after dosing because of invasiveness, which means that in most cases no full PK curves are available.

For performing a lavage on mechanically ventilated patients, a double catheter is inserted into the endotracheal tube and advanced blindly until resistance is met. Then a saline solution is instilled and collected by suction. This method is called mini-BAL and was used in 16 articles for sampling ELF and subsequently measuring drug concentrations (Supplementary section S2; Supplementary Table S1; 8, 141–155).

#### Bronchoscopic microsampling and bronchoabsorption

Two newer methods that sample ELF during a bronchoscopy are bronchoscopic microsampling (BMS) and bronchoabsorption. These are very similar, but the respective devices were developed and commercialized by different parties.^52–54^

The device, consisting of an outer polyethene tube and an inner cotton (BMS) or synthetic (bronchoabsorption) probe, is inserted through the bronchoscope and gently placed against the bronchial wall. After the inner probe has absorbed the fluid, the device is withdrawn, and the inner probe is placed in a tube. The probe is weighed before and after dilution to determine the volume of ELF recovered.^53^ BMS was used to sample ELF in eight articles (Supplementary section S2; Supplementary Table S1; 158–165). Bronchoabsorption was used in one article (Supplementary section S2; Supplementary Table S1; 56). Whereas fluid is collected from both alveolar and bronchial regions with BAL, specifically bronchial ELF is obtained with BMS.^55,56^

Both devices can be used multiple times at different sites in the respiratory tract. Additionally, they do not require instillation of saline, which makes them less complex to perform^53^ and less invasive than BAL. Recently, researchers developed a third device for sampling ELF in this manner with an inner probe of cellulose.^52^ To our knowledge, no studies have been conducted to compare the performance of all three devices.

#### Endotracheal suction and aspiration

A selection of 11 articles reported the collection of ‘bronchial secretions’. For this review, all studies that reported collecting bronchial/tracheal secretions by suction/aspiration were classified into the ELF sampling category, as was one study that reported collecting bronchial mucus with a catheter. Five articles reported collecting secretions without specifying the procedures, making categorization difficult. The other six studies reporting collection of bronchial secretions used endotracheal suction on mechanically ventilated patients (Supplementary section S2; Supplementary Table S1; 166, 167, 170–173).

### Microdialysis

Microdialysis is a method for monitoring concentrations of molecules, including drugs, in the interstitial fluid (ISF) over time. It can be performed in real time on tissue of patients (*in vivo* microdialysis) or on resected tissue (*ex vivo* microdialysis).

*In vivo* microdialysis is performed at the end of open chest surgery, and was used in nine articles (Supplementary section S2; Supplementary Table S1; 177–185). A probe with a semi-permeable membrane is inserted through the chest wall into the pulmonary tissue. Subsequently, the probe is perfused with a physiological solution and molecules in the ISF diffuse into the probe.^57–59^ The dialysate is collected and analysed. The retrodialysis method, which assumes the diffusion process is the same in both directions, is performed for calibration in each participant.^58–61^

For *ex vivo* microdialysis, lung tissue is removed during surgery. Directly after resection, the probe is inserted into the tissue, which is then perfused with a solution containing the drug of interest. This method was used in three articles (Supplementary section S2; Supplementary Table S1; 186–188) and these used the no-net flux method for calibration. A drug concentration range is infused and the separate dialysates are analysed.^62–64^ When the drug concentrations in perfusate and ISF are equal, there is no change in concentration in the outflowing dialysate. This is the point of no-net flux. These values are used to calculate the drug concentration in ISF.^65,66^

Both microdialysis approaches are very invasive due to the required surgery. In addition, the execution of the procedures and the calibration are complex. In spite of the complexity, microdialysis can provide a complete PK curve in human tissue over time.

## Measurement of intrapulmonary drug concentrations

In the reviewed studies, drug quantification occurred through LC-MS/MS in 67 studies; HPLC with varying modes of detection [e.g. fluorescence (*n* = 27) or UV (*n* = 33)] in 87 studies; diffusion bioassays in 25 studies; ligand binding/immunoassay in 11 studies; Positron emission tomography (PET) in 12 studies and MALDI-MS in three studies (see Supplementary section S2). Overall, limited assay development and validation data were published for the applied bio-analytical methods. This section will focus on the state-of-the-art analytical technology.

### Liquid chromatography

Bio-analytical methods typically consist of analyte extraction from biological samples, liquid chromatography to separate the analytes of interest from other drugs/metabolites and endogenous compounds in the matrix, and finally analyte detection. Detection is often done using tandem mass spectrometers, but classically also in the form of UV or fluorescence detection. The EMA and FDA have a synchronized guideline that provides recommendations for the validation of bio-analytical methods for chemical and biological drug quantification and their application in the analysis of study samples.^67,68^ Deviations from the recommendations may be acceptable with appropriate scientific justification.

Calibration standards (CSs) are used to determine the drug concentration of a sample with an unknown concentration. CSs as well as quality control samples (QCs) are prepared by spiking the blank biological matrix of interest with known concentrations of the analytes of interest. QCs are measured to assess the validity of the bio-analytical method and to determine accuracy and precision. CSs and QCs should be prepared from separate stock solutions. In many articles identified in this study, essential details on CS and QC preparation and assay validity are lacking. First, the calibration range should be reported, with a lower and higher limit of quantification. Second, accuracy and precision of the QCs should be reported. Indeed, precision was the most frequently reported validation parameter, often in the form of between- and within-day or within-assay precision. Accuracy was often not reported, but whether this is an omission in reporting or an indication that validation of the respective assays was lacking is unknown. It may also relate to the fact that in case of tissue bio-analysis, a true accuracy cannot be determined with spiked blank matrix. This is because spiked samples do not provide information on the drug recovery from the true site of disease, they are only markers of the extraction recovery of spiked matrix. One should consider evaluating precision and recovery of authentic non-blank tissues/cells, and checking the results for inconsistency.

Bio-analytical methods using site of disease matrices often face insufficient amount of available blank matrix (e.g. BAL fluid, lung tissue) to prepare CSs and QCs. To minimize the use of biological matrix, one could consider preparing and spiking a limited number of blank matrix QCs and validate them against a calibration curve in plasma or a solvent if there is no matrix effect.

The use of suitable internal standard (IS) can be critical in the development of a bio-analytical method. An IS should be added to all CSs, QCs and study samples during sample processing.^67,68^ The IS corrects for different sources of volume errors, including injection-to-injection variation, volume errors in sample preparation, and accounts for routine variations in the response of the chromatographic system. When using LC-MS/MS for quantitative assays, the use of a stable isotope labelled IS (SIL-IS) of each analyte is most suitable to also normalize e.g. for suppression or enhancement of ionization in the ion source and matrix effects. Of the 67 identified articles that used LC-MS/MS, only 36 reported use of an IS.

### MALDI-MS

Matrix-assisted laser desorption ionization mass spectrometry (MALDI-MS) imaging is an imaging technology that allows the creation of qualitative maps of a compound in tissue.^69–71^ Tissue sections are coated with a matrix that extracts molecules from tissue to aid in ionization. Subsequently, the molecules are ionized with a UV-laser and analysed with mass spectrometry. By generating a spectrum for every *x*,*y* coordinate of the tissue section, an image is created.^69,70^ As this method does not disrupt the tissue itself, the sections can be stained afterwards and merged with the molecular image.

Although qualitative maps by MALDI-MS provide relevant insights into local drug distribution without compromising tissue integrity, extracting quantitative information from these images is essential for accurately assessing drug concentrations in tissue.^71^ Only three articles identified in this review used MALDI-MS to quantify drug concentrations in human pulmonary tissue (Supplementary section S2; Supplementary Table S1; 23,32,34). This low number is not surprising, as this method is costly and requires lung tissue sections from patients. In addition, quantitative analysis of compounds with MALDI-MS is complex due to factors such as ion suppression and poor ionization.^70,71^ Nonetheless, it has been successfully applied in both human and animal lung tissue.^72–75^ Further development of this technology could be helpful in studying intrapulmonary drug concentrations with high special resolution.

### PET

PET is a molecular imaging technology widely used in clinical practice for evaluating organs and tissues of patients. After labelling the drug of interest with a radioactive tracer and intravenous administration to the participant, the tracer emits positive electrons. When these positrons collide with an electron in the tissue, this results in the release of two photons,^76^ which are detected by the PET camera to create images. The strength of the signal is related to the amount of drug molecules in tissue, thus providing quantitative information about tissue drug concentrations.^77,78^

PET was used for studying PK in the lungs of human participants in 12 articles (Supplementary section S2; Supplementary Table S2), with scans taken at maximum 8 hours after injection. All these studies applied correction for the attenuation effect of different tissues in the body.^79,80^ There was large variation in parameters reported to reflect the amount of drug molecules in the lungs measured by PET, showing the lack of consensus around this type of methodology. PET cannot distinguish parent compounds and metabolites,^81^ and most PET isotopes have a short half-life (e.g. 18F 110 min, 11C 20 min), which limits the time window for imaging.^76^ In addition, making PET labelled drugs, developing an injection fluid or infusion that is suitable for administration in humans, and performing PET itself are expensive.^82^ Despite these limitations, PET is well-suited for studying intrapulmonary concentrations in humans because it is non-invasive.

## Describing and predicting intrapulmonary pharmacokinetics with computational mathematical models

Mathematical models are used to describe and predict intrapulmonary concentrations of individual drugs and can also be applied to evaluate different treatment regimens. In this section we focus on population pharmacokinetic models (POPPK) and PBPK models, because these approaches were reported by most articles. Two articles reported the respective development and validation of a model for predicting ELF-plasma concentration ratio of antibiotics based on their chemical structure instead of based on PK (Supplementary section S2; Supplementary Table S5; 252, 253).

### Population pharmacokinetic models

POPPK models describe the behaviour of a drug in the body over time and are based on drug concentrations observed in participants. A POPPK model consists of three parts: a structural, stochastic and covariate model. The structural model describes the relationship between time and drug concentration for the typical individual in the population using a series of compartments. The stochastic model describes the variability in the observed population and usually consists of the variability between individuals and residual variability. Finally, the covariate model describes the effect of specific factors, such as sex and weight, on PK parameters. POPPK models are developed with non-linear mixed effects methodology.

POPPK modelling was used in 32 articles to describe and predict intrapulmonary PK in humans (Supplementary section S2; Supplementary Table S3). Most models were developed based on drug concentration measured in ELF (27 articles), but some used concentration data measured in lung tissue (three articles). One study linked a plasma POPPK model to a tissue model representing TB granulomas and using drug concentrations from animal experiments. Another study built a POPPK model with rabbit tissue data and simulated a humanized regimen.^13^ After model development, stochastic simulations can be performed to assess target attainment in plasma and lung tissue.

Generally, a plasma PK model is developed first and subsequently the intrapulmonary PK model is added, in most cases a single compartment (25 articles that represented either the ELF or lung tissue depending on the data). In a few articles, the intrapulmonary model was formed by two compartments representing the ELF and AMs (three articles). Finally, extensive POPPK models with three or more lung compartments were identified (three articles). These are of special interest because they are focused on TB, and compartments represented both healthy lung and pulmonary granulomas.

Three ways to model an intrapulmonary compartment were identified. The first and most common approach was to add a compartment with bidirectional mass transfer, similar to plasma compartment(s) (17 articles). The second approach is modelling an effect compartment, which works with a hypothetical link without mass transfer (six articles). The underlying assumption is that the drug concentration in intrapulmonary compartment(s) depends on the concentration in plasma, but the concentration in the central compartment is not meaningfully affected.^83^ Third, intrapulmonary concentrations can be modelled using a constant penetration ratio (five articles). This means that the intrapulmonary concentration is always assumed to be the same fraction of the plasma concentration.^83,84^

POPPK allows simultaneous modelling of plasma and intrapulmonary concentrations in all participants and can include several levels of stochastic variability. It is a well-known, relatively simple approach that makes optimal use of sampled sparse data. However, POPPK models are still inherently data-driven and, therefore, require the input of measured intrapulmonary concentration data.

### Physiologically based pharmacokinetic models

PBPK modelling is a mechanistic ‘bottom-up’ approach for characterizing and predicting pharmacokinetics *in vivo*. PBPK models integrate *a priori* information about anatomy, physiological processes and drug properties to predict the concentration of the drugs in plasma or tissue over time. A PBPK model consists of two main parts: a system-specific and a drug-specific part. The body is divided into compartments that represent physiological entities (e.g. organs and tissues) and this system is described by the system-specific parameters (e.g. volume of tissue compartments). In addition, the drug-specific part incorporates information about the physicochemical properties of the compound (e.g. molecular weight and solubility), the partitioning of compound between plasma and tissue, and protein binding. After model development and potentially parameter estimation/optimization, drug profiles in plasma and tissues are simulated. Preferably, the simulated concentrations are validated against observed data.

PBPK modelling was used in 35 articles to predict intrapulmonary PK in humans (Supplementary section S2; Supplementary Table S4). Input parameters were obtained from literature or estimated from available data. When animal data was used for model development (20 articles), respective species were mice, rats, monkeys or calves. Scaling from animal to human PBPK was done by replacing certain physiological parameters (e.g. organ weight, blood flow) and PK parameters (e.g. clearance) by human values.

Representation of the lung with one compartment occurred most often (20 articles), but models with two (four articles), four (two articles) and five compartments (two articles) were found as well, see Table 1. One lung model had seven compartments (two lung compartments, five lysosome). Three extensive lung PBPK models were identified (Supplementary section S2; Supplementary Table S4; 218, 225, 227). They all included an ELF compartment and two also had an AM compartment (Supplementary section S2: Supplementary Table S4; 218, 225). Two PBPK models focused on TB. One consisted of two compartments representing healthy lung and lesion, the other had cellular lesion and caseum compartments on top of vascular, interstitial and intracellular compartments (Supplementary section S2; Supplementary Table S4; 229, 233).

**Table 1.** PBPK models with two to four compartments and their division of the lung

PBPK model	Number of compartments	Lung compartments
An *et al.* 2012	Two	Interstitial and intracellular
Liu *et al.* 2021	Two (without extension)	Interstitial and intracellular
Mehta *et al.* 2023	Two	Healthy lung and pulmonary lesion (TB)
Yao *et al.* 2020 (and update Zhang *et al.* 2020)	Two	Lung general, lung tissue
Fan et al 2021	Four	Vascular, blood cells, interstitial, intracellular
Mehta *et al.* 2024	Four	Vascular, blood cells, interstitial, intracellular
Sharma *et al.* 2023	Five	Vascular, endothelial endosomal, interstitial, epithelial endosomal, ELF
Karakitsios *et al.* 2024	Five	Vascular, interstitial, intracellular, cellular lesion, caseum (TB)

The main advantage of PBPK modelling is that it can be used for predicting intrapulmonary concentrations in humans based on prior knowledge from preclinical experiments only. Although some human data are required for model validation, there is no need for large-scale invasive sampling in participants.

## Discussion

Suboptimal drug concentrations are related to higher chances of treatment failure, relapse and emergence of resistance.^18,85–87^ Due to insufficient intrapulmonary anti-TB drug concentration data, current dosing strategies of individual drugs in TB treatment might be inadequate. Exposure–response evaluations are commonly based on plasma concentrations alone, and it is unknown to which degree inclusion of intrapulmonary concentrations provides additive value. However, it is expected that studying intrapulmonary PK in humans will improve our understanding regarding the antibacterial effect of individual drugs at the site of disease.

As anti-TB drugs are given in combination, it is also important to match them in an optimal regimen based on achieved exposures. This is evidenced by unfavourable treatment outcomes caused by combination of anti-TB drugs with different half-lives (PK mismatch) in plasma.^85,88,89^ In adherence research, specific patterns of nonadherence determined failure and relapse,^90,91^ which probably relates to the PK characteristics of the composing drugs as well. Clearly matching of anti-TB drugs is equally important at the intrapulmonary level. Collecting intrapulmonary PK data from human participants could support modelling and simulation methods for predicting efficacy of new regimens. In line with this, multiple *in vitro* potency assays for TB drugs were recently evaluated and a selection of assays reproducing intrapulmonary conditions, i.e. in macrophages and caseum, predicted efficacy in mice and humans.^92^

This review has summarized the methods for sampling, quantifying, describing and predicting intrapulmonary drug concentrations used in 253 studies research articles. We did not limit the searches to TB or anti-TB drugs, because the ultimate objective was to provide a complete overview of methodology that could be useful for future TB research. Nevertheless, studies with specific focus on intrapulmonary PK of anti-TB drugs in humans were identified.

Each of the methods for sampling, quantification, description and prediction of intrapulmonary drug concentrations has advantages and disadvantages, which are presented in Table 2. The various sampled compartments represent the TB site of disease, the pulmonary granuloma, to a different extent. For example, if drug concentrations in ELF samples obtained with BAL or BMS/bronchoabsorption are high, this does not automatically mean the drug also penetrates into the granuloma. BAL allows sampling of AMs, which play an important role in early TB infection, but migrate to the granulomas during the disease process.^24,93^ For these reasons, the extent to which information about drug concentrations gathered with ELF sampling actually represents concentrations in granulomas is unclear and, therefore, it might not be the best method. Although *in vivo* microdialysis might be too invasive for TB patients, ex vivo microdialysis is also informative and has been applied successfully for sampling ISF from lung tissue (including granulomas) and measuring anti-TB drugs in the samples. This method is more complex but worth considering.^62–64^ Very informative would be the application of methods that retain spatial information and quantify granuloma concentrations: LCM combined with LC-MS/MS, MALDI-MS and PET. These methods have also been used successfully in preclinical studies^11–16,41,73,74,77,78,94,95^ and should be considered in follow-up research, even though they are technically complicated and costly. Finally, PK modelling methods are useful for describing and predicting intrapulmonary concentrations, with the main advantage of PBPK modelling over POPPK modelling its ability to predict site of disease concentrations in humans based on preclinical experiments only. Although a fully mechanistic model is often not possible, and assumptions have to be made, PBPK models can still provide valuable mechanistic understanding of lung PK processes. POPPK, on the other hand, is indispensable for exposure–response assessments.

**Table 2.** Overview of sampling, measurement and description/prediction methods to assess intrapulmonary drug concentrations with advantages and disadvantages

	Method	Compartment	Advantage(s)	Disadvantage(s)
Sampling	Spontaneous expectoration	Sputum	relatively simple non-invasive	not everyone can produce sputum on the spot non-homogeneous substance
	Sputum induction	Sputum	relatively simple minimally invasive	non-homogeneous substance
	Biopsy during bronchoscopy	Bronchial mucosa lung tissue	bronchoscopy is well-known procedure	invasive
	Biopsy during surgery	Parenchymal lung tissue	possible to take TB granuloma tissue	very invasive
	BAL	ELF	well-known procedure fluid collected contains macrophages	invasive sensitive to technical errors
	BMS/bronchoabsorption	ELF	no instillation of fluid makes it less invasive than BAL	relatively unknown procedure no macrophages in collected fluid
	Microdialysis	Lung ISF	allows measurement of drug concentrations at target site over time	very invasive complex
Sample preparation	Homogenization	Lung tissue	simple procedure	cellular structures destroyed, no spatial information
	LCM (before LC-MS/MS)	Lung tissue	spatial information	more complex
Measurement/quantification	MALDI-MS	Lung tissue	spatial information about drug distribution possible to study granuloma tissue	expensive complex quantitative analysis can be challenging
	PET	Lung tissue	non-invasive spatial information about drug distribution	expensive complex lack of consensus about methodology
Description and prediction	POPPK	Various compartments	less complex than PBPK	data-driven, without clinical data no prediction in humans
	PBPK	Various compartments	prediction in humans possible without clinical data bottom-up approach	preferably validated with real-life data more complex than POPPK

This review has several limitations. First, the process of abstract screening, article selection and data extraction was performed by only one author (I.v.d.F.). We do not expect to have missed any eligible methods because of this approach. In addition, this systematic review has not been registered in PROSPERO, because it is methodological and does not have a direct clinical recommendation as outcome. Finally, we did not discuss activation of drugs through bacterial pathways. When interpreting site of disease concentration data, any drug-specific need for local bio-activation should be considered.

The articles included in this review showed large variation in documenting details regarding sampling, sample preparation, bio-analysis and analytical method validation. This lack of standardization can form a barrier for future reviews and meta-analyses on this topic. In summary, we performed a systematic review and provided an overview of methods that can be applied to evaluate intrapulmonary concentrations of anti-TB drugs in patients. Many of the identified methods are associated with considerable limitations such as invasiveness and costs. When selecting a sampling method, one must also bear in mind whether it provides an adequate representation of site of disease pharmacokinetics. In addition to the choice of method, precise documentation of procedures is essential when designing future clinical studies, as more standardization will ensure that collected data can be compared across studies and contribute to a better understanding of intrapulmonary pharmacokinetics of anti-TB drugs.

## Supplementary Material

dkaf274_Supplementary_Data

## Supplementary data

Tables S1 to S7 and Sections S1 to S3 are available as Supplementary data at *JAC* Online.
