# Supplementary material for: Studying intrapulmonary pharmacokinetics for tuberculosis treatment: a systematic review of methodology
Source: J Antimicrob Chemother. 2025 Aug 14;80(10):2597–608. doi: 10.1093/jac/dkaf274 (PMC12494137; doi:10.1093/jac/dkaf274)
Supplement: dkaf274_Supplementary_Data [file dkaf274_supplementary_data.docx]

*Studying intrapulmonary pharmacokinetics for tuberculosis treatment: a systematic review of methodology*

Supplementary materials

Index

[**Supplementary materials 1**: search strategies and inclusion/exclusion criteria 2](#_Toc202771966)

[1 Search strategies: general 2](#_Toc202771967)

[1.1 General search strategy Embase 2](#_Toc202771968)

[1.2 General search strategy Pubmed 3](#_Toc202771969)

[2 Search strategies: PBPK 4](#_Toc202771970)

[2.1 PBPK search strategy Embase 4](#_Toc202771971)

[2.2 PBPK search strategy Pubmed 4](#_Toc202771972)

[3 Inclusion and exclusion criteria 5](#_Toc202771973)

[3.1 For general search 5](#_Toc202771974)

[3.2 For PBPK search 5](#_Toc202771975)

[**Supplementary materials 2**: studies included in the review 6](#_Toc202771976)

[Table S1 Sampling studies 6](#_Toc202771977)

[Table S2 PET studies 15](#_Toc202771978)

[Table S3 POPPK studies 15](#_Toc202771979)

[Table S4 PBPK studies 17](#_Toc202771980)

[Table S5 other models 19](#_Toc202771981)

[**Supplementary materials 3**: Preferred reporting items for systematic review and meta-analysis (PRISMA) checklists 36](#_Toc202771982)

[Table S6 PRISMA 2020 Abstracts Checklist 36](#_Toc202771983)

[Table S7 PRISMA 2020 Checklist 37](#_Toc202771984)

# **Supplementary materials 1**: search strategies and inclusion/exclusion criteria

# 1 Search strategies: general

## 1.1 General search strategy Embase

1.

(lung* or pulmon* or intrapulmon* or bronch* or alveol*).mp.

2.

drug diffusion/ or drug disposition/ or drug distribution/ or drug penetration/ or drug tissue level/ or drug tumor level/ or drug concentration/ or (penetration or tissue level* or drug level* or tissue concentration* or drug concentration* or drug distribution or target site concentration*).ti,ab,kf.

3.

(sampl* or model* or (broncho-alveolar lavage or BAL or microdialysis or lung resection or lung surgery or MALDI or PET or PKPD or pharmacokinetic model or PK model)).ti,ab,kf.

4.

1 and 2 and 3

5.

limit 4 to (english language and yr="1990 - Current" and article and "humans only (removes records about animals)")

6.

animal model/ or in vitro/ or (animal or in vitro).ti,ab,kf.

7.

5 not 6

## 1.2 General search strategy Pubmed

(("lung"[MeSH Terms] OR "lung"[All Fields] OR "pulmon*"[All Fields] OR "intrapulmon*"[All Fields] OR "bronch*"[All Fields] OR "alveol*"[All Fields])

AND

("tissue distribution"[MeSH Terms] OR "drug diffusion"[Title/Abstract] OR "drug disposition"[Title/Abstract] OR "drug distribution"[Title/Abstract] OR ("drug"[Title/Abstract] AND"penetration"[Title/Abstract]) OR ("drug"[Title/Abstract] AND "tissue level"[Title/Abstract]) OR ("drug"[Title/Abstract] AND "tumor level"[Title/Abstract]))

AND

("sampl*"[Title/Abstract] OR "model*"[Title/Abstract] OR "broncho alveolar lavage"[Title/Abstract] OR "BAL"[Title/Abstract] OR "microdialysis"[Title/Abstract] OR "lung resection"[Title/Abstract] OR "lung surgery"[Title/Abstract] OR "MALDI"[Title/Abstract] OR "PET"[Title/Abstract] OR "PKPD"[Title/Abstract] OR "pharmacokinetic model"[Title/Abstract] OR "PK model"[Title/Abstract]))

NOT

("models, animal"[MeSH Terms] OR "in vitro techniques"[MeSH Terms] OR ("animal"[Title/Abstract] OR "in vitro"[Title/Abstract]))

Filters: English, from 1990 - 2023 Sort by: Most Recent

# 2 Search strategies: PBPK

## 2.1 PBPK search strategy Embase

1.

(lung* or pulmon* or intrapulmon* or bronch* or alveol*).mp.

2.

PBPK/ or PBPK.ti,ab,kf. or physiologically-based pharmacokinetic*.ti,ab,kf. or physiologically based pharmacokinetic*.ti,ab,kf.

3.

1 and 2

4.

limit 3 to (yr="1990 - 2023" and article)

5.

limit 4 to (english and yr="1990 -Current" and article)

## 2.2 PBPK search strategy Pubmed

("lung"[MeSH Terms] OR "lung"[All Fields] OR "pulmon*"[All Fields] OR "intrapulmon*"[All Fields] OR "bronch*"[All Fields] OR "alveol*"[All Fields])

AND ("PBPK"[Title/Abstract] OR "physiologically based pharmacokinetic model*"[All Fields] OR "physiologically based pharmacokinetic model*"[All Fields])

Filters: from 1990 - 2023 Sort by: Most Recent

# 3 Inclusion and exclusion criteria

## 3.1 For general search

***Inclusion criteria***

- Peer-reviewed research articles
- Studies in which intrapulmonary drug concentrations are

1. quantified/measured

AND/OR

2. described with *pharmacokinetic/pharmacological* models

- Data from human participants
  - alive, not after death
- English language
- Published since 1990

***Exclusion criteria***

- Studies in which only plasma concentrations are quantified or predicted
- Studies with only In vitro/cell experiments
- Localisation & imaging drug distribution (PET) data without quantitative drug concentration measurements
- Studies with quantification of compounds that are
  - Not drugs, such as endogenous compounds (proteins), nutrients, inflammation markers, environmental compounds
  - Non-organic drugs such as platinum
- Full articles that are not accessible

## 3.2 For PBPK search

***Inclusion criteria***

- Peer-reviewed research articles
- Studies where physiologically based pharmacokinetic model (PBPK) is used to predict intrapulmonary (drug) concentrations in humans, more specifically:
  - Measurements can be from animals or in silico data, but for inclusion predicted drug concentrations in humans must be reported
  - PBPK models of the lungs should be more than one compartment, OR can be one compartment when based on experiments in which animal or human tissue concentration data was collected.
- English language
- Published since 1990

***Exclusion criteria***

- All studies with inhalation as route of administration in animals and humans
- Studies where PBPK models were developed to evaluate drug-drug interactions
- Pregnancy PBPK models

# **Supplementary materials 2**: studies included in the review

Abbreviations

N.R. Not Reported

N.A. Not Applicable

ELF Epithelial Lining Fluid

AC Alveolar cells

ISF interstitial fluid

* classified as

# Table S1 Sampling studies

| **Article number** | **Author + year** | **Drug concentrations measured in** | **Sample collection** | **Analysis method** |
| --- | --- | --- | --- | --- |
| 1 | Begg et al. 2000 ^1^ | Sputum | N.R. | HPLC-fluorescence |
| 2 | Klekner et al. 2006 ^2^ | Sputum | Tracheal suction | Alternative |
| 3 | Kovarik et al. 1992 ^3^ | Sputum | N.R. | Diffusion bioassay |
| 4 | Moriarty et al. 2007 ^4^ | Sputum | Spontaneous expectoration | HPLC |
| 5 | Qin et al. 2022 ^5^ | Sputum | N.R. | HPLC-UV |
| 6 | Rubino et al. 2021 ^6^ | Sputum | Spontaneous expectoration or induction | LC-MS/MS |
| 7 | Wei et al. 2022 ^7^ | Sputum | N.R. | HPLC-UV |
| 8 | Baldwin et al. 1990 ^8^ | Sputum, bronchial mucosa tissue, ELF and AC | Sputum: N.R. Bronchoscopy with biopsy, BAL and mini-BAL | Diffusion bioassay |
| 9 | Watanabe et al. 2001 ^9^ | Sputum, parenchymal tissue | Sputum: N.R. , biopsy during lung surgery | HPLC |
| 10 | Baldwin et al. 1990 ^10^ | Bronchial mucosa tissue | Bronchoscopy with biopsy | Diffusion bioassay |
| 11 | Baldwin et al. 1990 ^11^ | Bronchial mucosa tissue, parenchymal tissue | Bronchoscopy with biopsy | Diffusion bioassay |
| 12 | Biancosino et al. 2007 ^12^ | Parenchymal (healthy and tumour), lymphnode tissue | Biopsy during lung surgery | HPLC-fluorescence |
| 13 | Birmingham et al. 1999 ^13^ | Parenchymal tissue | Biopsy during lung surgery | HPLC-fluorescence |
| 14 | Bohnenstengel et al. 2000 ^14^ | Parenchymal tissue | Biopsy during lung surgery | HPLC-UV |
| 15 | Breilh et al. 2001 ^15^ | Parenchymal tissue | Biopsy during lung surgery | HPLC-fluorescence |
| 16 | Byl et al. 1999 ^16^ | Bronchial mucosa tissue, parenchymal tissue | Biopsy during lung surgery | Diffusion bioassay |
| 17 | Cao et al. 2020 ^17^ | Bronchial mucosa tissue, parenchymal tissue | Biopsy during lung surgery | HPLC-fluorescence |
| 18 | Concia et al. 2005 ^18^ | Parenchymal tissue (healthy) | Biopsy during lung surgery | HPLC-UV |
| 19 | Dan et al. 1993 ^19^ | Bronchial mucosa tissue, parenchymal tissue | Biopsy during lung surgery | HPLC-fluorescence |
| 20^20^ | Dheda et al. 2018 ^20^ | Parenchymal tissue | Biopsy during lung surgery | LC-MS/MS |
| 21 | Esmailpour et al. 1997 ^21^ | Parenchymal tissue (peripheral) and bronchial tissue (central) both tumour | Biopsy during lung surgery | Ligand binding / immuno-assay |
| 22 | Fabre et al. 1991 ^22^ | Bronchial mucosa tissue, parenchymal tissue | Bronchoscopy with biopsy | HPLC-fluorescence |
| 23 | Fehniger et al. 2011 ^23^ | Bronchial mucosa tissue | Bronchoscopy with biopsy | MALDI-MSI |
| 24 | Gould et al. 1994 ^24^ | Bronchial mucosa tissue, parenchymal tissue | Bronchoscopy with biopsy | HPLC |
| 25 | Grootenboers et al. 2007 ^25^ | Parenchymal tissue, tumour tissue | Biopsy during lung surgery | HPLC-fluorescence |
| 26 | Haraguchi et al. 2007 ^26^ | Parenchymal tissue | Biopsy during lung surgery | HPLC |
| 27 | Lankheet et al. 2015 ^27^ | Parenchymal tissue (tumour) | Biopsy during lung surgery | LC-MS/MS |
| 28 | Lee et al. 1998 ^28^ | Parenchymal tissue (healthy) | Biopsy during lung surgery | HPLC-fluorescence |
| 29 | Leveque et al. 1993 ^29^ | Parenchymal tissue (healthy and tumour) | Biopsy during lung surgery | HPLC-fluorescence |
| 30 | Naline et al. 1991 ^30^ | Parenchymal tissue (healthy and tumour), bronchial and arterial wall tissue, bronchial mucus collected with catheter | Biopsy during lung surgery | HPLC |
| 31 | Phillips et al. 2022 ^31^ | Parenchymal tissue (tumour-adjacent uninvolved tissue) | Biopsy during lung surgery | LC-MS/MS |
| 32 | Prideaux et al. 2015 ^32^ | Parenchymal tissue (healthy and lesion) | Biopsy during lung surgery | LC-MS/MS, MALDI-MSI |
| 33 | Serour et al. 1991 ^33^ | Parenchymal tissue | Biopsy during lung surgery | Diffusion bioassay |
| 34 | Strydom et al. 2019 ^34^ | Parenchymal tissue (9 distinct human lesion-specific tissues) | Biopsy during lung surgery | LC-MS/MS and MALDI-MSI |
| 35 | Weidekamm et al. 1993 ^35^ | Parenchymal tissue | Biopsy during lung surgery | HPLC-fluorescence |
| 36 | Andrews et al. 1995 ^36^ | Bronchial mucosa tissue, ELF and AC | Bronchoscopy with biopsy and BAL | Diffusion bioassay |
| 37 | Andrews et al. 1997 ^37^ | Bronchial mucosa tissue, ELF and AC | Bronchoscopy with biopsy and BAL | Diffusion bioassay |
| 38 | Andrews et al. 2003 ^38^ | Bronchial mucosa tissue, ELF and AC | Bronchoscopy with biopsy and BAL | Diffusion bioassay |
| 39 | Andrews et al. 2007 ^39^ | Bronchial mucosa tissue, ELF and AC | Bronchoscopy with biopsy and BAL | Diffusion bioassay |
| 40 | Baldwin et al. 1993 ^40^ | Bronchial mucosa tissue, ELF and AC | Bronchoscopy with biopsy and BAL | Diffusion bioassay |
| 41 | Burkhardt et al. 2005 ^41^ | Parenchymal tissue, ELF | Biopsy during lung surgery and bronchoscopy with BAL | HPLC |
| 42 | Cook et al. 1994 ^42^ | Bronchial mucosa tissue, ELF and AC | Bronchoscopy with biopsy and BAL | Diffusion bioassay |
| 43 | Danesi et al. 2003 ^43^ | Parenchymal tissue, ELF | Biopsy during lung surgery and bronchoscopy with BAL | Diffusion bioassay |
| 44 | Di Paolo et al. 2002 ^44^ | Parenchymal tissue, ELF | Biopsy during lung surgery and BAL through endotracheal tube | HPLC-fluorescence |
| 45 | Honeybourne et al. 1994 ^45^ | Bronchial mucosa tissue, ELF and AC | Bronchoscopy with biopsy and BAL | HPLC |
| 46 | Honeybourne et al. 2003 ^46^ | Bronchial mucosa tissue, ELF and AC | Bronchoscopy with biopsy and BAL | HPLC-UV |
| 47 | Khair et al. 2001 ^47^ | Bronchial mucosa tissue, ELF and AC | Bronchoscopy with biopsy and BAL | Diffusion bioassay |
| 48 | Krumpe et al. 1999 ^48^ | Parenchymal tissue, ELF* | Bronchoscopy with transbronchial biopsy and BAL, aspiration | HPLC-UV |
| 49 | Lappin et al. 2013 ^49^ | Bronchial mucosa tissue, ELF and AC | Bronchoscopy with biopsy and BAL | HPLC |
| 50 | Leroyer et al. 1998 ^50^ | Bronchial mucosa tissue, ELF* | Bronchoscopy with biopsy, aspiration | Diffusion bioassay |
| 51 | Lopez-Varela et al. 2022 ^51^ | Endobronchial lymph node tissue, ELF | Endobronchial biopsy, bronchoscopy with BAL | LC-MS/MS |
| 52 | Lucchi et al. 2008 ^52^ | Parenchymal tissue, ELF and AC | Biopsy during lung surgery and bronchoscopy with BAL | HPLC |
| 53 | Marra et al. 2002 ^53^ | Parenchymal tissue, ELF | Bronchoscopy with transbronchial biopsy and BAL | HPLC-UV |
| 54 | Matera et al. 1997 ^54^ | Bronchial mucosa tissue, ELF* | Bronchoscopy with biopsy and BAL, aspiration | Diffusion bioassay |
| 55 | Muller-Sereys et al. 1992 ^55^ | Parenchymal tissue, ELF | Biopsy during lung surgery and bronchoscopy with BAL | Diffusion bioassay |
| 56 | Sadiq et al. 2021 ^56^ | Bronchial mucosa tissue, epithelial tissue, ELF | Bronchoscopy with BAL and mucosal biopsy and brush biopsy, bronchosorption | LC-MS/MS |
| 57 | Soman et al. 1999 ^57^ | Bronchial mucosa tissue, ELF and AC | Bronchoscopy with biopsy and BAL | Diffusion bioassay |
| 58 | Wildfeuer et al. 1994 ^58^ | Bronchial mucosa tissue, ELF | Bronchoscopy with biopsy and BAL | Diffusion bioassay and GC-MS |
| 59 | Ziglam et al. 2002 ^59^ | Bronchial mucosa tissue, ELF and AC | Bronchoscopy with biopsy and BAL | Diffusion bioassay |
| 60 | Allegranzi et al. 2000 ^60^ | ELF | Bronchoscopy with BAL | HPLC-UV |
| 61 | Andersen et al. 2017 ^61^ | ELF | Bronchoscopy with BAL | LC-MS/MS |
| 62 | Asempa et al. 2023 ^62^ | ELF | Bronchoscopy with BAL | LC-MS/MS |
| 63 | Begg et al. 2019 ^63^ | ELF | Bronchoscopy with BAL | LC-MS/MS |
| 64 | Benitez-Cano et al. 2020 ^64^ | ELF | Bronchoscopy with BAL | HPLC-UV |
| 65 | Bergmann 2024 ^65^ | ELF | Bronchoscopy with BAL | LC-MS/MS |
| 66 | Capitano et al. 2004 ^66^ | ELF and AC | Bronchoscopy with BAL | HPLC-fluorescence |
| 67 | Capitano et al. 2006 ^67^ | ELF | Bronchoscopy with BAL | LC-MS/MS |
| 68 | Carcas et al. 1999 ^68^ | ELF | Bronchoscopy with BAL | Ligand binding / immuno-assay |
| 69 | Caro et al. 2020 ^69^ | ELF | Bronchoscopy with BAL | LC-MS/MS |
| 70 | Cassidy et al. 2011 ^70^ | ELF | Bronchoscopy with BAL | LC-MS/MS and Ligand binding / immuno-assay |
| 71 | Chandokar et al. 2012 ^71^ | ELF | Bronchoscopy with BAL | LC-MS/MS |
| 72 | Connors et al. 2014 ^72^ | ELF and AC | Bronchoscopy with BAL | LC-MS/MS |
| 73 | Conte et al. 1995 ^73^ | ELF and AC | Bronchoscopy with BAL | HPLC and Ligand binding / immuno-assay |
| 74 | Conte et al. 1996 ^74^ | ELF and AC | Bronchoscopy with BAL | HPLC-fluorescence |
| 75 | Conte et al. 1999 ^75^ | ELF and AC | Bronchoscopy with BAL | HPLC |
| 76 | Conte et al. 2000 (1) ^76^ | ELF and AC | Bronchoscopy with BAL | HPLC |
| 77 | Conte et al. 2000 (2) ^77^ | ELF and AC | Bronchoscopy with BAL | LC-MS/MS |
| 78 | Conte et al. 2001 ^78^ | ELF and AC | Bronchoscopy with BAL | LC-MS/MS |
| 79 | Conte et al. 2002 (1) ^79^ | ELF and AC | Bronchoscopy with BAL | HPLC |
| 80 | Conte et al. 2002 (2) ^80^ | ELF and AC | Bronchoscopy with BAL | LC-MS/MS |
| 81 | Conte et al. 2004 (1) ^81^ | ELF and AC | Bronchoscopy with BAL | HPLC-UV |
| 82 | Conte et al. 2004 (2) ^82^ | ELF and AC | Bronchoscopy with BAL | HPLC-fluorescence |
| 83 | Conte et al. 2004 (3) ^83^ | ELF and AC | Bronchoscopy with BAL | LC-MS/MS |
| 84 | Conte et al. 2005 (1) ^84^ | ELF and AC | Bronchoscopy with BAL | LC-MS/MS |
| 85 | Conte et al. 2005 (2) ^85^ | ELF and AC | Bronchoscopy with BAL | LC-MS/MS |
| 86 | Conte et al. 2006 ^86^ | ELF and AC | Bronchoscopy with BAL | LC-MS/MS |
| 87 | Conte et al. 2009 ^87^ | ELF and AC | Bronchoscopy with BAL | LC-MS/MS |
| 88 | Conte, Lin, Zurlinden 2000 (1) ^88^ | ELF and AC | Bronchoscopy with BAL | HPLC-UV |
| 89 | Conte, Lin, Zurlinden 2000 (2) ^89^ | ELF and AC | Bronchoscopy with BAL | HPLC-fluorescence |
| 90 | Cousson et al. 2015 ^90^ | ELF | Bronchoscopy with BAL | HPLC-UV |
| 91 | Crandon et al. 2009 ^91^ | ELF and AC | Bronchoscopy with BAL | LC-MS/MS |
| 92 | Cruciani et al. 1997 ^92^ | ELF | Bronchoscopy with BAL | HPLC-UV |
| 93 | Das et al. 2020 ^93^ | ELF | Bronchoscopy with BAL | LC-MS/MS |
| 94 | Dimopoulos et al. 2022 ^94^ | ELF | Bronchoscopy with BAL | HPLC-UV |
| 95 | Furuie et al. 2010 ^95^ | ELF and AC | Bronchoscopy with BAL | LC-MS/MS |
| 96 | Furuie et al. 2018 ^96^ | ELF and AC | Bronchoscopy with BAL | HPLC-fluorescence |
| 97 | Gotfried et al. 2001 ^97^ | ELF and AC | Bronchoscopy with BAL | HPLC-fluorescence |
| 98 | Gotfried et al. 2003 ^98^ | ELF and AC | Bronchoscopy with BAL | HPLC |
| 99 | Gotfried et al. 2017 ^99^ | ELF and AC | Bronchoscopy with BAL | LC-MS/MS |
| 100 | Housman et al. 2012 ^100^ | ELF and AC | Bronchoscopy with BAL | LC-MS/MS |
| 101 | Huang et al. 2014 ^101^ | ELF | Bronchoscopy with BAL | LC-MS/MS |
| 102 | Husain et al. 2010 ^102^ | ELF | Bronchoscopy with BAL | HPLC-UV |
| 103 | Imberti et al. 2010 ^103^ | ELF | Bronchoscopy with BAL | HPLC and Ligand binding / immuno-assay |
| 104 | Kadota et al. 2002 ^104^ | ELF and AC | Bronchoscopy with BAL | Diffusion bioassay |
| 105 | Katsube et al. 2019 ^105^ | ELF and AC | Bronchoscopy with BAL | LC-MS/MS |
| 106 | Katsube et al. 2021 ^106^ | ELF | Bronchoscopy with BAL | LC-MS/MS |
| 107 | Lamer et al. 1993 ^107^ | ELF | Bronchoscopy with BAL | HPLC |
| 108 | Lodise et al. 2008 (1) ^108^ | ELF | Bronchoscopy with BAL | LC-MS/MS |
| 109 | Lodise et al. 2008 (2) ^109^ | ELF | Bronchoscopy with BAL | LC-MS/MS |
| 110 | Lodise et al. 2011 (1) ^110^ | ELF | Bronchoscopy with BAL | LC-MS/MS |
| 111 | Lodise et al. 2011 (2) ^111^ | ELF | Bronchoscopy with BAL | LC-MS/MS |
| 112 | Luyt et al. 2011 ^112^ | ELF | Bronchoscopy with BAL | Ligand binding / immuno-assay |
| 113 | McCallum et al. 2021 ^113^ | ELF and AC | Bronchoscopy with BAL | LC-MS/MS |
| 114 | Mendivil et al. 2012 ^114^ | ELF | Bronchoscopy with BAL | Ligand binding / immuno-assay |
| 115 | Muller-Sereys et al. 2001 ^115^ | ELF and AC | Bronchoscopy with BAL | Diffusion bioassay |
| 116 | Najmeddin et al. 2020 ^116^ | ELF | Bronchoscopy with BAL | Ligand binding / immuno-assay |
| 117 | Nicasio et al. 2009 ^117^ | ELF and AC | Bronchoscopy with BAL | HPLC |
| 118 | Nicolau et al. 2012 ^118^ | ELF | Bronchoscopy with BAL | HPLC |
| 119 | Nicolau et al. 2015 ^119^ | ELF | Bronchoscopy with BAL | LC-MS/MS |
| 120 | O'Brien et al. 1998 ^120^ | ELF | Bronchoscopy with BAL | HPLC-UV |
| 121 | Olsen et al. 1996 ^121^ | ELF and AC | Bronchoscopy with BAL | HPLC |
| 122 | Ong et al. 2005 ^122^ | ELF and AC | Bronchoscopy with BAL | HPLC |
| 123 | Paiboonvong et al. 2019 ^123^ | ELF | Bronchoscopy with BAL | LC-MS/MS |
| 124 | Panidis et al. 2005 ^124^ | ELF | Bronchoscopy with BAL | Ligand binding / immuno-assay |
| 125 | Patel et al. 1996 ^125^ | ELF and AC | Bronchoscopy with BAL | HPLC |
| 126 | Riccobene et al. 2016 ^126^ | ELF | Bronchoscopy with BAL | LC-MS/MS |
| 127 | Rizk et al. 2018^127^ | ELF and AC | Bronchoscopy with BAL | LC-MS/MS |
| 128 | Rodvold et al. 1997 ^128^ | ELF and AC | Bronchoscopy with BAL | HPLC |
| 129 | Rodvold et al. 2003 ^129^ | ELF and AC | Bronchoscopy with BAL | HPLC-fluorescence |
| 130 | Rodvold et al. 2012 ^130^ | ELF and AC | Bronchoscopy with BAL | LC-MS/MS |
| 131 | Rodvold et al. 2017 ^131^ | ELF and AC | Bronchoscopy with BAL | LC-MS/MS |
| 132 | Rodvold et al. 2018 (1) ^132^ | ELF and AC | Bronchoscopy with BAL | LC-MS/MS |
| 133 | Rodvold et al. 2018 (2) ^133^ | ELF and AC | Bronchoscopy with BAL | LC-MS/MS |
| 134 | Rodvold et al 2024 ^134^ | ELF and AM | Bronchoscopy with BAL | LC-MS/MS |
| 135 | Shelton et al. 2011 ^135^ | ELF | Bronchoscopy with BAL | LC-MS/MS |
| 136 | Tayman et al. 2011 ^136^ | ELF | Bronchoscopy with BAL | HPLC |
| 137 | Tenero et al. 2013 ^137^ | ELF and AC | Bronchoscopy with BAL | LC-MS/MS |
| 138 | Twigg et al. 2010 ^138^ | ELF | Bronchoscopy with BAL | HPLC-UV |
| 139 | Walsh et al. 2010 ^139^ | ELF and AC | Bronchoscopy with BAL | HPLC-UV |
| 140 | Wenzler et al. 2015 ^140^ | ELF and AC | Bronchoscopy with BAL | LC-MS/MS |
| 141 | Zeitlinger et al. 2016 ^141^ | ELF | Bronchoscopy with BAL | LC-MS/MS |
| 142 | Zhang et al. 2010 ^142^ | ELF | Bronchoscopy with BAL | HPLC-fluorescence |
| 143 | Athanassa et al. 2012 ^143^ | ELF | Mini-BAL | HPLC-UV |
| 144 | Boisson et al. 2014 ^144^ | ELF | Mini-BAL | LC-MS/MS |
| 145 | Boselli et al. 2003 ^145^ | ELF | Mini-BAL | HPLC-UV |
| 146 | Boselli et al. 2004 (1) ^146^ | ELF | Mini-BAL | HPLC-UV |
| 147 | Boselli et al. 2004 (2) ^147^ | ELF | Mini-BAL | HPLC-UV |
| 148 | Boselli et al. 2005 (1) ^148^ | ELF | Mini-BAL | HPLC-UV |
| 149 | Boselli et al. 2005 (2) ^149^ | ELF | Mini-BAL | HPLC-UV |
| 150 | Boselli et al. 2006 ^150^ | ELF | Mini-BAL | HPLC-UV |
| 151 | Boselli et al. 2007 ^151^ | ELF | Mini-BAL | LC-MS/MS |
| 152 | De Pascale et al. 2020 ^152^ | ELF | Mini-BAL | LC-MS/MS |
| 153 | Felton et al. 2014 ^153^ | ELF | Mini-BAL | LC-MS/MS |
| 154 | Gkoufa et al. 2022 ^154^ | ELF | Mini-BAL | LC-MS/MS |
| 155 | Layios et al. 2022 ^155^ | ELF | Mini-BAL | LC-MS/MS |
| 156 | Toutain et al. 2004^156^ | ELF | Mini-BAL | HPLC-UV |
| 157 | Wu et al. 2022 ^157^ | ELF | Mini-BAL | HPLC-UV |
| 158 | Funatsu et al. 2014 ^158^ | ELF | Bronchoscopy with BMS | LC-MS/MS |
| 159 | Funatsu et al. 2016 ^159^ | ELF | Bronchoscopy with BMS | LC-MS/MS |
| 160 | Hasegawa et al. 2009 ^160^ | ELF | Bronchoscopy with BMS | LC-MS/MS |
| 161 | Kikuchi et al. 2007 (1) ^161^ | ELF | Bronchoscopy with BMS and BAL | HPLC-fluorescence |
| 162 | Kikuchi et al. 2007 (2) ^162^ | ELF | Bronchoscopy with BMS and BAL | Diffusion bioassay |
| 163 | Kikuchi et al. 2007 (3) ^163^ | ELF and AC | Bronchoscopy with BMS and BAL | LC-MS/MS |
| 164 | Kikuchi et al. 2009 ^164^ | ELF | Bronchoscopy with BMS and BAL | LC-MC/MS and HPLC-fluorescence |
| 165 | Yamazaki et al. 2003 ^165^ | ELF | Bronchoscopy with BMS | LC-MS/MS and HPLC |
| 166 | Bergogne-Bérézin et al. 1992 ^166^ | ELF* | N.R. | Diffusion bioassay |
| 167 | Bergogne-Bérézin et al. 1994 ^167^ | ELF* | N.R. | Diffusion bioassay |
| 168 | Jehl et al. 1994 ^168^ | ELF* | Through endotracheal tube or with tracheostomy cannula with trap | HPLC-UV |
| 169 | Kontou et al. 2011 ^169^ | ELF* | Endotracheal suction (with mucus aspirator through endotracheal tube) | HPLC-fluorescence |
| 170 | Leone et al. 2004 ^170^ | ELF* | Bronchoscopy with protected catheter, protected catheter/specimen brush, or BAL | HPLC-fluorescence |
| 171 | Monforte et al. 2003 ^171^ | ELF* | Bronchoscopy with bronchial aspirated secretions and BAL | HPLC-UV |
| 172 | Rebholz et al 2024 ^172^ | ELF* | Endotracheal suction | LC-MS/MS |
| 173 | Santré et al. 1995 ^173^ | ELF* | Endotracheal suction | Ligand binding / immuno-assay |
| 174 | Saux et al. 1994 ^174^ | ELF* | Endotracheal suction (with mucus aspirator through endotracheal tube) | HPLC-UV |
| 175 | Simon et al. 2003 ^175^ | ELF* | Endotracheal suction (with mucus aspirator through endotracheal or tracheostomy tube) | HPLC-fluorescence |
| 176 | Valcke et al. 1992 ^176^ | ELF* | Bronchoscopy with collection of endotracheal aspirated secretions and BAL | Ligand binding / immuno-assay |
| 177 | Edlinger-Stanger et al. 2021 ^177^ | Lung ISF | In vivo microdialysis | HPLC-UV |
| 178 | Herkner et al. 2002 ^178^ | Lung ISF | In vivo microdialysis | HPLC |
| 179 | Hutschala et al. 2005 ^179^ | Lung ISF | In vivo microdialysis | HPLC-fluorescence |
| 180 | Hutschala et al. 2008 ^180^ | Lung ISF | In vivo microdialysis | HPLC-fluorescence |
| 181 | Lindenmann et al. 2011 ^181^ | Lung ISF | In vivo microdialysis | HPLC-UV |
| 182 | Matzi et al. 2010 ^182^ | Lung ISF | In vivo microdialysis | Alternative |
| 183 | Tomaselli et al. 2003 ^183^ | Lung ISF | In vivo microdialysis | HPLC-UV |
| 184 | Tomaselli et al. 2004 ^184^ | Lung ISF | In vivo microdialysis | HPLC |
| 185 | Zeitlinger et al. 2007 ^185^ | Lung ISF | In vivo microdialysis | HPLC-fluorescence |
| 186 | Heinrichs et al. 2018 ^186^ | Lung ISF | Ex vivo microdialysis | LC-MS/MS |
| 187 | Kempker et al. 2015 ^187^ | Lung ISF | Ex vivo microdialysis | LC-MS/MS |
| t188 | Kempker et al. 2017 ^188^ | Lung ISF | Ex vivo microdialysis | LC-MS/MS |

# Table S2 PET studies

| Article number | Author +year | Method | Attenuation correction | PK parameter reported (with unit) |
| --- | --- | --- | --- | --- |
| 189 | Brunner et al. 2004 ^189^ | PET | Transmission scan | Cmax, Tmax, t1/2, and AUCtot |
| 190 | Fishman et al. 1993 (1) ^190^ | PET | Transmission scan | Concentration in microgram/gram |
| 191 | Fishman et al. 1993 (2) ^191^ | PET | Transmission scan | AUC in microgram/gram |
| 192 | Fishman et al. 1996 ^192^ | PET | Transmission scan | AUC in microgram/gram |
| 193 | Fischman et al. 1998 ^193^ | PET | Transmission scan | AUC in microgram/gram |
| 194 | Garg et al. 2017 ^194^ | PET-CT | CT | Uptake μSv/MBq |
| 195 | Harris et al. 2013 ^195^ | PET-CT | CT | Uptake in maxSUV |
| 196 | Ordonez et al. 2020 ^196^ | PET-CT | Tissue density [X-ray attenuation value (Hounsfield Unit)] | Tissue-to-plasma AUC ratio for each lesion |
| 197 | Van der Veldt et al. 2011 ^197^ | PET-CT | CT | Net influx rate in mL·cm−3·min−1 |
| 198 | Van der Veldt et al. 2013 ^198^ | PET-CT | CT | AUC tumor in kBq·minute·mL−1 |
| 199 | Volkow et al. 1992 ^199^ | PET | Emission scan | Uptake in % dose/cc |
| 200 | Volkow et al. 2010 ^200^ | PET | Emission scan | Uptake in %Dose/cc |

# Table S3 POPPK studies

| **Article number** | **Author + year** | **Drug concentrations described/predicted in…** | **Nr of lung compartments** | **Lung compartment(s)** | **Type of lung compartment** |
| --- | --- | --- | --- | --- | --- |
| 15 | Breilh et al. 2001 | Lung tissue | One | Lung | Normal |
| 201 | Cicchese et al. 2020 ^201^ | TB granulomas | N.A | Tissue model (special) | Plasma PK linked to agent-based environment through blood vessels placed on the simulation grid |
| 202 | Clewe et al. 2015 ^202^ | ELF and ACs (BAL) | Two | ELF and AC | Effect |
| 93 | Das et al. 2020 | ELF (BAL) | One | ELF | Normal |
| 94 | Dimopoulos et al. 2022 | ELF (BAL) | One | ELF | Constant penetration coefficient |
| 203 | Drusano et al. 2002 ^203^ | ELF (BAL) | One | ELF | Normal |
| 204 | Drusano et al. 2011 ^204^ | ELF (BAL) | One | ELF | Normal |
| 153 | Felton et al. 2014 | ELF (mini-BAL) | One | ELF | Normal |
| 205 | Felton et al. 2018 ^205^ | ELF (mini-BAL) from previous studies | One | ELF | Normal |
| 154 | Gkoufa et al. 2022 ^154^ | ELF (mini-BAL) | One | ELF | Normal |
| 206 | Goutelle et al. 2009 ^206^ | ELF and ACs (BAL) | Two | ELF and AC | Normal |
| 207 | Hughes et al. 2020 ^207^ | Lung tissue and more (biopsy) | Two | Lung tissue and AM cytosol | Unclear |
| 208 | Ikawa et al. 2014 ^208^ | ELF (BMS) | One | ELF | Normal |
| 209 | Kawaguchi et al. 2022 ^209^ | ELF (BAL) | One | ELF | Effect |
| 210 | Kuti, Nicolau 2015 ^210^ | ELF (BAL) | One | ELF | Normal |
| 155 | Layios et al. 2022 | ELF (mini-BAL) | One | ELF | Normal |
| 108 | Lodise et al. 2008 (1) | ELF (BAL) | One | ELF | Normal |
| 109 | Lodise et al. 2008 (2) | ELF (BAL) | One | ELF | Normal |
| 110 | Lodise et al. 2011 (1) | ELF (BAL) | One | ELF | Normal |
| 111 | Lodise et al. 2011 (2) | ELF (BAL) | One | ELF | Normal |
| 51 | Lopez-Variela et al. 2022 | ELF (BAL) & lymph node tissue (biopsy) | More than two | Lung and lesion | Effect |
| 113 | McCallum et al. 2021 | ELF and AMs (BAL) | Two | ELF and AM | Constant penetration coefficient |
| 126 | Riccobene et al. 2016 | ELF(BAL) | One | ELF | Constant penetration coefficient |
| 127 | Rizk et al. 2018 | ELF (BAL) | One | ELF | Constant penetration coefficient |
| 211 | Rubino et al. 2007 ^211^ | ELF (BAL) | One | Lung | Normal |
| 212 | Shorr et al. 2021 ^212^ | ELF | One | ELF | Effect |
| 175 | Simon et al. 2003 ^175^ | ELF* | One | ELF | Normal |
| 34 | Strydom et al. 2019 | Lung tissue (biopsy) | More than two | Lung and lesion | Effect |
| 213 | Van Hasselt et al. 2016 ^213^ | ELF (BAL) | One | ELF | Constant penetration coefficient |
| 214 | Xiao et al. 2016 ^214^ | ELF (BAL) | One | ELF | Normal |
| 215 | Zimmerman et al. 2017 ^215^ | Lung tissue (biopsy) from rats | Three | Lung, cellular lesion, caseous lesion (TB) | Effect |
| 216 | Zhang et al. 2021 ^216^ | ELF (BAL) | One | ELF | Effect |

# Table S4 PBPK studies

| **Article number** | **Author + year** | **Drug concentration data from...** | **Species (experiments)** | **Nr of lung compartments** | **Lung compartments** |
| --- | --- | --- | --- | --- | --- |
| 217 | An et al. 2012 ^217^ | Lung tissue (biopsy) | Mice | Two | Interstitial and intracellular |
| 218 | Aulin et al. 2022 ^218^ | N.A. | N.A. | At least 18 | Three models, the simplest one with blood, ELF and AM compartments for six lung segments |
| 219 | Bae et al. 2019 ^219^ | Lung tissue (biopsy) | Mice | One | Lung |
| 220 | Chao et al. 2023 ^220^ | Lung tissue (biopsy) | Mice and rats | One | Lung |
| 221 | Cui et al. 2020 ^221^ | N.A. | N.A. | 28 | See Gaohua et al. 2015 |
| 222 | Fan et al. 2022 ^222^ | Lung tissue | Monkey | Four | Vascular, blood cells, interstitial, and intracellular |
| 223 | Fu et al. 2019 ^223^ | Lung tissue (biopsy) | Mice | One | Lung |
| 224 | Gao et al. 2019 ^224^ | Lung tissue (biopsy) | Rats | One | Lung |
| 225 | Gaohua et al. 2015 ^225^ | N.A. | N.A. | 28 | Five lung segments (right lung low, top, middle lobe and left lung low and top lobe) and two airway segments (upper and lower), with each blood, mass, fluid and alveoli compartments |
| 226 | Humphries et al. 2021 ^226^ | N.A. | N.A. | 28 | See Gaohua et al. 2015 |
| 227 | Jagdale et al. 2022 ^227^ | ELF (BAL) | Humans | 18 (three compartments with each six subcompartments) | Alveolar, upper airway, lower airway (with each vascular, endothelial endosomal, interstitial, epithelial endosomal, ELF and airway space) |
| 228 | Jermain et al. 2020 ^228^ | Lung tissue (biopsy) | Calves (lung tissue), humans (plasma) | One | Lung |
| 229 | Karakitsios et al. 2024 ^229^ | Lung tissue | Rabbits, mice and humans | Five | Vascular, interstitial, intracellular, cellular lesion, caseum |
| 230 | Lee et al. 2020 ^230^ | Lung tissue | Mice | One | Lung |
| 231 | Liu, Jusko 2021 ^231^ | Lung tissue from rats (biopsy) | Rats (plasma&lung tissue) and humans (plasma) | Two (base) and seven (lysosome) | Interstitial & total cytosol (base), cytosol, neutral lipids and more (lysosome) |
| 232 | Martins et al. 2023 ^232^ | Lung ISF (microdialysis) | Humans | One | Lung |
| 233 | Mehta et al. 2023 ^233^ | Lung tissue (biopsy) | Mice | Two | Lung and lesion (effect compartments) |
| 234 | Mehta et al. 2024 ^234^ | Plasma | Humans (patients) | Four | Vascular, blood cells, interstitial, and intracellular |
| 235 | Muliatadan et al. 2022 ^235^ | Lung tissue (biopsy) | Mice | One | Lung |
| 236 | Rowland Yeo et al. 2020 ^236^ | N.A. | N.A. | 28 | See Gaohua et al. 2015 |
| 237 | Salerno et al. 2017 ^237^ | ELF (BAL) | Humans (patients) | One | ELF |
| 238 | Sharma et al. 2023 ^238^ | ELF (BAL) | Mice | Five | Vascular, endothelial endosomal, interstitial, epithelial endosomal and ELF |
| 239 | Shibata et al. 2021 ^239^ | Lung tissue (biopsy) | Rats | One | Lung |
| 241 | Shin et al. 2004 ^240^ | Lung tissue (biopsy) | Rats | One | Lung |
| 241 | Shin et al. 2009 ^241^ | Lung tissue (biopsy) | Rats | One | Lung |
| 242 | Shin et al. 2011 ^242^ | Lung tissue (biopsy) | Rats | One | Lung |
| 243 | Sun et al. 2015 ^243^ | Lung tissue (biopsy) | Rats | One | Lung |
| 244 | Themans et al. 2019 ^244^ | ELF (BAL) | Humans (patients) | One | Lung |
| 245 | Wyska et al. 2016 ^245^ | Lung tissue (biopsy) | Mice | One | Lung |
| 246 | Yang et al. 2023 ^246^ | Lung tissue (biopsy) | Mice and hamsters | One | Lung |
| 247 | Yao et al. 2020 ^247^ | N.A. | N.A. | Two | Subcompartments |
| 248 | Zang et al. 2018 ^248^ | Lung tissue (biopsy) | Mice | One | Lung |
| 249 | Zhang et al. 2020 ^249^ | N.A. | N.A. | Two | Subcompartments |
| 250 | Zhu et al. 2022 ^250^ | N.A. | N.A. | One | Lung |
| 251 | Zurlinden et al. 2016 ^251^ | Lung tissue (biopsy) | Rats (plasma & lung tissue) | One | Lung |

# Table S5 other models

| **Article number** | **Author + year** | **Model type** | **Drug concentrations described/predicted in…** |
| --- | --- | --- | --- |
| 252 | Valitano et al. 2016 ^252^ | Structure based regression | ELF |
| 253 | Aulin et al. 2018 ^253^ | Structure based regression | ELF |

1. Begg EJ, Robson RA, Saunders DA*, et al.* The pharmacokinetics of oral fleroxacin and ciprofloxacin in plasma and sputum during acute and chronic dosing. *Br J Clin Pharmacol* 2000; **49**: 32-8. <https://doi.org/10.1046/j.1365-2125.2000.00105.x>

2. Klekner A, Bagyi K, Bognar L*, et al.* Effectiveness of cephalosporins in the sputum of patients with nosocomial bronchopneumonia. *J Clin Microbiol* 2006; **44**: 3418-21. <https://doi.org/10.1128/JCM.00893-06>

3. Kovarik JM, Hoepelman AIM, Smit JM*, et al.* Steady-state pharmacokinetics and sputum penetration of lomefloxacin in patients with chronic obstructive pulmonary disease and acute respiratory tract infections. *Antimicrob Agents Chemother* 1992; **36**: 2458-61. <https://doi.org/10.1128/aac.36.11.2458>

4. Moriarty TF, McElnay JC, Elborn JS*, et al.* Sputum antibiotic concentrations: implications for treatment of cystic fibrosis lung infection. *Pediatr Pulmonol* 2007; **42**: 1008-17. <https://doi.org/10.1002/ppul.20671>

5. Qin X, Kong L, Wu C*, et al.* Pharmacokinetic/pharmacoddynamic analysis of high-dose tigecycline, by Monte Carlo simulation, in plasma and sputum of patients with hospital-acquired pneunomia *J Clin Pharm Ther* 2022; **47**: 2312-9. <https://doi.org/10.1111/jcpt.13823>

6. Rubino CM, Onufrak NJ, van Ingen J*, et al.* Population Pharmacokinetic Evaluation of Amikacin Liposome Inhalation Suspension in Patients with Treatment-Refractory Nontuberculous Mycobacterial Lung Disease. *Eur J Drug Metab Pharmacokinet* 2021; **46**: 277-87. <https://doi.org/10.1007/s13318-020-00669-7>

7. Wei Y, Zhang H, Fu M*, et al.* Plasma and Intrapulmonary Pharmacokinetics, and Dosage Regimen Optimization of Linezolid for Treatment of Gram-Positive Cocci Infections in Patients with Pulmonary Infection After Cerebral Hemorrhage. *Infection and Drug Resistance* 2022; **15**: 1733-42. <https://doi.org/10.2147/IDR.S357300>

8. Baldwin DR, Wise R, Andrews JM*, et al.* Azithromycin concentrations at the sites of pulmonary infection. *Eur Respir J* 1990; **3**: 886-90.

9. Watanabe A, Anzai Y, Niitsuma K*, et al.* Penetration of minocycline hydrochloride into lung tissue and sputum. *Chemotherapy* 2001; **47**: 1-9. <https://doi.org/10.1159/000048494>

10. Baldwin DR, Honeybourne D, Andrews JM*, et al.* Concentrations of oral lomefloxacin in serum and bronchial mucosa. *Antimicrob Agents Chemother* 1990; **34**: 1017-9. <https://doi.org/10.1128/aac.34.6.1017>

11. Baldwin DR, Wilkinson L, Andrews JM*, et al.* Concentrations of temafloxacin in serum and bronchial mucosa. *Eur J Clin Microbiol Infect Dis* 1990; **9**: 432-4. <https://doi.org/10.1007/bf01979477>

12. Biancosino C, Albert M, Linder A. Acute toxicity of irinotecan in the ex-vivo isolated perfused human lung model--high-dose therapy during isolated perfusion without acute toxic edema. *Interact Cardiovasc Thorac Surg* 2007; **6**: 583-7. <https://doi.org/10.1510/icvts.2007.152165>

13. Birmingham MC, Guarino R, Heller A*, et al.* Ciprofloxacin concentrations in lung tissue following a single 400 mg intravenous dose. *J Antimicrob Chemother* 1999; **43**: 43-8. <https://doi.org/10.1093/jac/43.suppl_1.43>

14. Bohnenstengel F, Friedel G, Ritter CA*, et al.* Variability of cyclosphosphamide uptake into human bronchial carcinoma: Consequences for local bioactivation. *Cancer Chemother Pharmacol* 2000; **45**: 63-8. <https://doi.org/10.1007/PL00006745>

15. Breilh D, Saux MC, Maire P*, et al.* Mixed pharmacokinetic population study and diffusion model to describe ciprofloxacin lung concentrations. *Comput Biol Med* 2001; **31**: 147-55. <https://doi.org/10.1016/S0010-4825(00)00031-7>

16. Byl B, Jacobs F, Roucloux I*, et al.* Penetration of meropenem in lung, bronchial mucosa, and pleural tissues. *Antimicrob Agents Chemother* 1999; **43**: 681-2. <https://doi.org/10.1128/aac.43.3.681>

17. Cao G, Zhu Y, Xie X*, et al.* Pharmacokinetics and pharmacodynamics of levofloxacin in bronchial mucosa and lung tissue of patients undergoing pulmonary operation. *Exp Ther Med* 2020; **20**: 607-16. <https://doi.org/10.3892/etm.2020.8715>

18. Concia E, Allegranzi B, Ciottoli GB*, et al.* Penetration of orally administered prulifloxacin into human lung tissue. *Clin Pharmacokinet* 2005; **44**: 1287-94. <https://doi.org/10.2165/00003088-200544120-00007>

19. Dan M, Torossian K, Weissberg D*, et al.* The penetration of ciprofloxacin into bronchial mucosa, lung parenchyma, and pleural tissue after intravenous administration. *Eur J Clin Pharmacol* 1993; **44**: 101-2. <https://doi.org/10.1007/BF00315290>

20. Dheda K, Lenders L, Magombedze G*, et al.* Drug-penetration gradients associated with acquired drug resistance in patients with tuberculosis. *Am J Respir Crit Care Med* 2018; **198**: 1208-19. <https://doi.org/10.1164/rccm.201711-2333OC>

21. Esmailpour N, Hogger P, Rabe KF*, et al.* Distribution of inhaled fluticasone propionate between human lung tissue and serum in vivo. *Eur Respir J* 1997; **10**: 1496-9. <https://doi.org/10.1183/09031936.97.10071496>

22. Fabre D, Bressolle F, Gomeni R*, et al.* Steady-state pharmacokinetics of ciprofloxacin in plasma from patients with nosocomial pneumonia: Penetration of the bronchial mucosa. *Antimicrob Agents Chemother* 1991; **35**: 2521-5. <https://doi.org/10.1128/aac.35.12.2521>

23. Fehniger TE, Végvári A, Rezeli M*, et al.* Direct demonstration of tissue uptake of an inhaled drug: proof-of-principle study using matrix-assisted laser desorption ionization mass spectrometry imaging. *Anal Chem* 2011; **83**: 8329-36. <https://doi.org/10.1021/ac2014349>

24. Gould IM, Harvey G, Golder D*, et al.* Penetration of amoxycillin/clavulanic acid into bronchial mucosa with different dosing regimens. *Thorax* 1994; **49**: 999-1001. <https://doi.org/10.1136/thx.49.10.999>

25. Grootenboers MJJH, Hendriks JMH, Van Boven WJ*, et al.* Pharmacokinetics of isolated lung perfusion with melphalan for resectable pulmonary metastases, a phase I and extension trial. *J Surg Oncol* 2007; **96**: 583-9. <https://doi.org/10.1002/jso.20838>

26. Haraguchi S, Hioki M, Yamashita K*, et al.* Ciprofloxacin penetration into the pulmonary parenchyma in Japanese patients. *Surg Today* 2007; **37**: 282-4. <https://doi.org/10.1007/s00595-006-3393-4>

27. Lankheet NA, Schaake EE, Burgers SA*, et al.* Concentrations of Erlotinib in Tumor Tissue and Plasma in Non-Small-Cell Lung Cancer Patients After Neoadjuvant Therapy. *Clin Lung Cancer* 2015; **16**: 320-4. <https://doi.org/10.1016/j.cllc.2014.12.012>

28. Lee LJ, Sha X, Gotfried MH*, et al.* Penetration of levofloxacin into lung tissue after oral administration to subjects undergoing lung biopsy or lobectomy. *Pharmacotherapy* 1998; **18**: 35-41. <https://doi.org/10.1002/j.1875-9114.1998.tb03824.x>

29. Levêque D, Quoix E, Dumont P*, et al.* Pulmonary distribution of vinorelbine in patients with non-small-cell lung cancer. *Cancer Chemother Pharmacol* 1993; **33**: 176-8. <https://doi.org/10.1007/bf00685338>

30. Naline E, Sanceaume M, Toty L*, et al.* Penetration of minocycline into lung tissues. *Br J Clin Pharmacol* 1991; **32**: 402-4. <https://doi.org/10.1111/j.1365-2125.1991.tb03920.x>

31. Phillips JD, Pooler DB, Ness DB*, et al.* Tumour, whole-blood, plasma and tissue concentrations of metformin in lung cancer patients. *Br J Clin Pharmacol* 2022; **89**: 1027-35. <https://doi.org/10.1111/bcp.15546>

32. Prideaux B, Via LE, Zimmerman MD*, et al.* The association between sterilizing activity and drug distribution into tuberculosis lesions. *Nat Med* 2015; **21**: 1223-7. <https://doi.org/10.1038/nm.3937>

33. Serour F, Dan M, Gorea A*, et al.* Penetration of ofloxacin into human lung tissue following a single oral dose of 200 milligrams. *Antimicrob Agents Chemother* 1991; **35**: 380-1. <https://doi.org/10.1128/AAC.35.2.380>

34. Strydom N, Gupta SV, Fox WS*, et al.* Tuberculosis drugs' distribution and emergence of resistance in patient's lung lesions: A mechanistic model and tool for regimen and dose optimization. *PLoS Med* 2019; **16**: e1002773. <https://doi.org/10.1371/journal.pmed.1002773>

35. Weidekamm E, Portmann R. Penetration of fleroxacin into body tissues and fluids. *Am J Med* 1993; **94**: 75s-80s. <https://doi.org/10.1016/S0002-9343(20)31142-6>

36. Andrews JM, Wise R, Baldwin DR*, et al.* Concentrations of ceftibuten in plasma and the respiratory tract following a single 400 mg oral dose. *Int J Antimicrob Agents* 1995; **5**: 141-4. <https://doi.org/10.1016/0924-8579(94)00044-u>

37. Andrews JM, Honeybourne D, Jevons G*, et al.* Concentrations of levofloxacin (HR 355) in the respiratory tract following a single oral dose in patients undergoing fibre-optic bronchoscopy. *J Antimicrob Chemother* 1997; **40**: 573-7. <https://doi.org/10.1093/jac/40.4.573>

38. Andrews J, Honeybourne D, Jevons G*, et al.* Concentrations of garenoxacin in plasma, bronchial mucosa, alveolar macrophages and epithelial lining fluid following a single oral 600 mg dose in healthy adult subjects. *J Antimicrob Chemother* 2003; **51**: 727-30. <https://doi.org/10.1093/jac/dkg110>

39. Andrews J, Honeybourne D, Ashby J*, et al.* Concentrations in plasma, epithelial lining fluid, alveolar macrophages and bronchial mucosa after a single intravenous dose of 1.6 mg/kg of icaprim (AR-100) in healthy men. *J Antimicrob Chemother* 2007; **60**: 677-80. <https://doi.org/10.1093/jac/dkm242>

40. Baldwin DR, Wise R, Andrews JM*, et al.* Comparative bronchoalveolar concentrations of ciprofloxacin and lomefloxacin following oral administration. *Respir Med* 1993; **87**: 595-601. <https://doi.org/10.1016/s0954-6111(05)80262-8>

41. Burkhardt O, Majcher-Peszynska J, Borner K*, et al.* Penetration of ertapenem into different pulmonary compartments of patients undergoing lung surgery. *J Clin Pharmacol* 2005; **45**: 659-65. <https://doi.org/10.1177/0091270005276117>

42. Cook PJ, Andrews JM, Woodcock J*, et al.* Concentration of amoxycillin and clavulanate in lung compartments in :Adults without pulmonary infection. *Thorax* 1994; **49**: 1134-8. <https://doi.org/10.1136/thx.49.11.1134>

43. Danesi R, Lupetti A, Barbara C*, et al.* Comparative distribution of azithromycin in lung tissue of patients given oral daily doses of 500 and 1000 mg. *J Antimicrob Chemother* 2003; **51**: 939-45. <https://doi.org/10.1093/jac/dkg138>

44. Di Paolo A, Barbara C, Chella A*, et al.* Pharmacokinetics of azithromycin in lung tissue, bronchial washing, and plasma in patients given multiple oral doses of 500 and 1000 mg daily. *Pharmacol Res* 2002; **46**: 545-50. <https://doi.org/10.1016/s1043661802002384>

45. Honeybourne D, Kees F, Andrews JM*, et al.* The levels of clarithromycin and its 14-hydroxy metabolite in the lung. *Eur Respir J* 1994; **7**: 1275-80. <https://doi.org/10.1183/09031936.94.07071275>

46. Honeybourne D, Tobin C, Jevons G*, et al.* Intrapulmonary penetration of linezolid. *J Antimicrob Chemother* 2003; **51**: 1431-4. <https://doi.org/10.1093/jac/dkg262>

47. Khair OA, Andrews JM, Honeybourne D*, et al.* Lung concentrations of telithromycin after oral dosing. *J Antimicrob Chemother* 2001; **47**: 837-40. <https://doi.org/10.1093/jac/47.6.837>

48. Krumpe P, Lin CC, Radwanski E*, et al.* The penetration of ceftibuten into the respiratory tract. *Chest* 1999; **116**: 369-74. <https://doi.org/10.1378/chest.116.2.369>

49. Lappin G, Boyce MJ, Matzow T*, et al.* A microdose study of 14C-AR-709 in healthy men: Pharmacokinetics, absolute bioavailability and concentrations in key compartments of the lung. *Eur J Clin Pharmacol* 2013; **69**: 1673-82. <https://doi.org/10.1007/s00228-013-1528-2>

50. Leroyer C, Muller-Serieys C, Quiot JJ*, et al.* Dirithromycin concentrations in bronchial mucosa and secretions. *Respiration* 1998; **65**: 381-5. <https://doi.org/10.1159/000029299>

51. Lopez-Varela E, Abulfathi AA, Strydom N*, et al.* Drug concentration at the site of disease in children with pulmonary tuberculosis. *J Antimicrob Chemother* 2022; **77**: 1710-9. <https://doi.org/10.1093/jac/dkac103>

52. Lucchi M, Damle B, Fang A*, et al.* Pharmacokinetics of azithromycin in serum, bronchial washings, alveolar macrophages and lung tissue following a single oral dose of extended or immediate release formulations of azithromycin. *J Antimicrob Chemother* 2008; **61**: 884-91. <https://doi.org/10.1093/jac/dkn032>

53. Marra F, Partovi N, Wasan KM*, et al.* Amphotericin B disposition after aerosol inhalation in lung transplant recipients. *Ann Pharmacother* 2002; **36**: 46-51. <https://doi.org/10.1345/aph.1A015>

54. Matera MG, Tufano MA, Polverino M*, et al.* Pulmonary concentrations of dirithromycin and erythromycin during acute exacerbation of mild chronic obstructive pulmonary disease. *Eur Respir J* 1997; **10**: 98-103. <https://doi.org/10.1183/09031936.97.10010098>

55. Muller-Serieys C, Bancal C, Dombret MC*, et al.* Penetration of cefpodoxime proxetil in lung parenchyma and epithelial lining fluid of noninfected patients. *Antimicrob Agents Chemother* 1992; **36**: 2099-103. <https://doi.org/10.1128/AAC.36.10.2099>

56. Sadiq MW, Holz O, Ellinghusen BD*, et al.* Lung pharmacokinetics of inhaled and systemic drugs: A clinical evaluation. *Br J Pharmacol* 2021; **178**: 4440-51. <https://doi.org/10.1111/bph.15621>

57. Soman A, Honeybourne D, Andrews J*, et al.* Concentrations of moxifloxacin in serum and pulmonary compartments following a single 400 mg oral dose in patients undergoing fibre-optic bronchoscopy. *J Antimicrob Chemother* 1999; **44**: 835-8. <https://doi.org/10.1093/jac/44.6.835>

58. Wildfeuer A, Rühle KH, Bölcskei PL*, et al.* Concentrations of ampicillin and sulbactam in serum and in various compartments of the respiratory tract of patients. *Infection* 1994; **22**: 149-51. <https://doi.org/10.1007/bf01739027>

59. Ziglam HM, Baldwin DR, Daniels I*, et al.* Rifampicin concentrations in bronchial mucosa, epithelial lining fluid, alveolar macrophages and serum following a single 600 mg oral dose in patients undergoing fibre-optic bronchoscopy. *J Antimicrob Chemother* 2002; **50**: 1011-5. <https://doi.org/10.1093/jac/dkf214>

60. Allegranzi B, Cazzadori A, Di Perri G*, et al.* Concentrations of single-dose meropenem (1 g iv) in bronchoaveolar lavage and epithelial lining fluid. *J Antimicrob Chemother* 2000; **46**: 319-22. <https://doi.org/10.1093/jac/46.2.319>

61. Andersen CU, Sonderskov LD, Bendstrup E*, et al.* Voriconazole Concentrations in Plasma and Epithelial Lining Fluid after Inhalation and Oral Treatment. *Basic and Clinical Pharmacology and Toxicology* 2017; **121**: 430-4. <https://doi.org/10.1111/bcpt.12820>

62. Asempa TE, Kuti JL, Nascimento JC*, et al.* Bronchopulmonary disposition of IV cefepime/taniborbactam (2-0.5 g) administered over 2 h in healthy adult subjects. *J Antimicrob Chemother* 2023; **78**: 703-9. <https://doi.org/10.1093/jac/dkac447>

63. Begg M, Wilson R, Hamblin JN*, et al.* Relationship between pharmacokinetics and pharmacodynamic responses in healthy smokers informs a once-daily dosing regimen for nemiralisib. *J Pharmacol Exp Ther* 2019; **369**: 337-44. <https://doi.org/10.1124/jpet.118.255109>

64. Benitez-Cano A, Luque S, Sorli L*, et al.* Intrapulmonary concentrations of meropenem administered by continuous infusion in critically ill patients with nosocomial pneumonia: A randomized pharmacokinetic trial. *Critical Care* 2020; **24** 55. <https://doi.org/10.1186/s13054-020-2763-4>

65. Bergmann F, Wolfl-Duchek M, Jorda A*, et al.* Pharmacokinetics of isavuconazole at different target sites in healthy volunteers after single and multiple intravenous infusions. *J Antimicrob Chemother* 2024; **79**: 1169-75. <https://doi.org/10.1093/jac/dkae088>

66. Capitano B, Mattoes HM, Shore E*, et al.* Steady-state intrapulmonary concentrations of moxifloxacin, levofloxacin, and azithromycin in older adults. *Chest* 2004; **125**: 965-73. <https://doi.org/10.1378/chest.125.3.965>

67. Capitano B, Potoski BA, Husain S*, et al.* Intrapulmonary penetration of voriconazole in patients receiving an oral prophylactic regimen. *Antimicrob Agents Chemother* 2006; **50**: 1878-80. <https://doi.org/10.1128/aac.50.5.1878-1880.2006>

68. Carcas AJ, Garcia-Satue JL, Zapater P*, et al.* Tobramycin penetration into epithelial lining fluid of patients with pneumonia. *Clin Pharmacol Ther* 1999; **65**: 245-50. <https://doi.org/10.1016/S0009-9236(99)70103-7>

69. Caro L, Nicolau DP, De Waele JJ*, et al.* Lung penetration, bronchopulmonary pharmacokinetic/pharmacodynamic profile and safety of 3 g of ceftolozane/tazobactam administered to ventilated, critically ill patients with pneumonia. *J Antimicrob Chemother* 2020; **75**: 1546-53. <https://doi.org/10.1093/jac/dkaa049>

70. Cassidy JP, Amin N, Marino M*, et al.* Insulin lung deposition and clearance following technosphere insulin inhalation powder administration. *Pharm Res* 2011; **28(9)**: 2157-64. <https://doi.org/10.1007/s11095-011-0443-4>

71. Chandorkar G, Huntington JA, Gotfried MH*, et al.* Intrapulmonary penetration of ceftolozane/tazobactam and piperacillin/tazobactam in healthy adult subjects. *J Antimicrob Chemother* 2012; **67**: 2463-9. <https://doi.org/10.1093/jac/dks246>

72. Connors KP, Housman ST, Pope JS*, et al.* Phase I, open-label, safety and pharmacokinetic study to assess bronchopulmonary disposition of intravenous eravacycline in healthy men and women. *Antimicrob Agents Chemother* 2014; **58**: 2113-8. <https://doi.org/10.1128/AAC.02036-13>

73. Conte JE, Jr., Golden JA, Duncan S*, et al.* Intrapulmonary pharmacokinetics of clarithromycin and of erythromycin. *Antimicrob Agents Chemother* 1995; **39**: 334-8. <https://doi.org/10.1128/aac.39.2.334>

74. Conte JE, Jr., Golden J, Duncan S*, et al.* Single-dose intrapulmonary pharmacokinetics of azithromycin, clarithromycin, ciprofloxacin, and cefuroxime in volunteer subjects. *Antimicrob Agents Chemother* 1996; **40**: 1617-22. <https://doi.org/10.1128/aac.40.7.1617>

75. Conte JE, Jr., Golden JA, Duncan S*, et al.* Intrapulmonary concentrations of pyrazinamide. *Antimicrob Agents Chemother* 1999; **43**: 1329-33. <https://doi.org/10.1128/aac.43.6.1329>

76. Conte Jr JE, Golden JA, McQuitty M*, et al.* Single-dose intrapulmonary pharmacokinetics of rifapentine in normal subjects. *Antimicrob Agents Chemother* 2000; **44**: 985-90. <https://doi.org/10.1128/aac.44.4.985-990.2000>

77. Conte JE, Jr., Golden JA, McQuitty M*, et al.* Effects of AIDS and gender on steady-state plasma and intrapulmonary ethionamide concentrations. *Antimicrob Agents Chemother* 2000; **44**: 1337-41. <https://doi.org/10.1128/aac.44.5.1337-1341.2000>

78. Conte JE, Jr., Golden JA, Kipps J*, et al.* Effects of AIDS and gender on steady-state plasma and intrapulmonary ethambutol concentrations. *Antimicrob Agents Chemother* 2001; **45**: 2891-6. <https://doi.org/10.1128/aac.45.10.2891-2896.2001>

79. Conte Jr JE, Golden JA, McQuitty M*, et al.* Effects of gender, AIDS, and acetylator status on intrapulmonary concentrations of isoniazid. *Antimicrob Agents Chemother* 2002; **46**: 2358-64. <https://doi.org/10.1128/AAC.46.8.2358-2364.2002>

80. Conte JE, Jr., Golden JA, Kipps J*, et al.* Intrapulmonary pharmacokinetics of linezolid. *Antimicrob Agents Chemother* 2002; **46**: 1475-80. <https://doi.org/10.1128/aac.46.5.1475-1480.2002>

81. Conte JE, Jr., Golden JA, Kipps J*, et al.* Intrapulmonary pharmacokinetics and pharmacodynamics of itraconazole and 14-hydroxyitraconazole at steady state. *Antimicrob Agents Chemother* 2004; **48**: 3823-7. <https://doi.org/10.1128/aac.48.10.3823-3827.2004>

82. Conte JE, Golden JA, Kipps JE*, et al.* Effect of sex and AIDS status on the plasma and intrapulmonary pharmacokinetics of rifampicin. *Clin Pharmacokinet* 2004; **43**: 395-404. <https://doi.org/10.2165/00003088-200443060-00003>

83. Conte JE, Jr., Golden JA, Kipps J*, et al.* Steady-state plasma and intrapulmonary pharmacokinetics and pharmacodynamics of cethromycin. *Antimicrob Agents Chemother* 2004; **48**: 3508-15. <https://doi.org/10.1128/aac.48.9.3508-3515.2004>

84. Conte JE, Jr., Golden JA, Kelly MG*, et al.* Steady-state serum and intrapulmonary pharmacokinetics and pharmacodynamics of tigecycline. *Int J Antimicrob Agents* 2005; **25**: 523-9. <https://doi.org/10.1016/j.ijantimicag.2005.02.013>

85. Conte Jr JE, Golden JA, Kelley MG*, et al.* Intrapulmonary pharmacokinetics and pharmacodynamics of meropenem. *Int J Antimicrob Agents* 2005; **26**: 449-56. <https://doi.org/10.1016/j.ijantimicag.2005.08.015>

86. Conte Jr JE, Golden JA, McIver M*, et al.* Intrapulmonary pharmacokinetics and pharmacodynamics of high-dose levofloxacin in healthy volunteer subjects. *Int J Antimicrob Agents* 2006; **28**: 114-21. <https://doi.org/10.1016/j.ijantimicag.2006.03.022>

87. Conte JE, Jr., Golden JA, Krishna G*, et al.* Intrapulmonary pharmacokinetics and pharmacodynamics of posaconazole at steady state in healthy subjects. *Antimicrob Agents Chemother* 2009; **53**: 703-7. <https://doi.org/10.1128/aac.00663-08>

88. Conte Jr JE, Lin E, Zurlinden E. High-performance liquid chromatographic determination of pyrazinamide in human plasma, bronchoalveolar lavage fluid, and alveolar cells. *J Chromatogr Sci* 2000; **38**: 33-7. <https://doi.org/10.1093/chromsci/38.1.33>

89. Conte Jr JE, Lin E, Zurlinden E. Liquid chromatographic determination of rifampin in human plasma, bronchoalveolar lavage fluid, and alveolar cells. *J Chromatogr Sci* 2000; **38**: 72-6. <https://doi.org/10.1093/chromsci/38.2.72>

90. Cousson J, Floch T, Guillard T*, et al.* Lung concentrations of ceftazidime administered by continuous versus intermittent infusion in patients with ventilator-associated pneumonia. *Antimicrob Agents Chemother* 2015; **59**: 1905-9. <https://doi.org/10.1128/AAC.04232-14>

91. Crandon JL, Banevicius MA, Fang AF*, et al.* Bronchopulmonary disposition of intravenous voriconazole and anidulafungin given in combination to healthy adults. *Antimicrob Agents Chemother* 2009; **53**: 5102-7. <https://doi.org/10.1128/aac.01042-09>

92. Cruciani M, Gatti G, Mengoli C*, et al.* Penetration of dapsone into pulmonary lining fluid of human immunodeficiency virus type 1-infected patients. *Antimicrob Agents Chemother* 1997; **41**: 1077-81. <https://doi.org/10.1128/aac.41.5.1077>

93. Das S, Fitzgerald R, Ullah A*, et al.* Intrapulmonary Pharmacokinetics of Cefepime and Enmetazobactam in Healthy Volunteers: Towards New Treatments for Nosocomial Pneumonia. *Antimicrob Agents Chemother* 2020; **65**: e01468-20. <https://doi.org/10.1128/AAC.01468-20>

94. Dimopoulos G, Almyroudi MP, Kapralos I*, et al.* Intrapulmonary pharmacokinetics of high doses of tigecycline in patients with ventilator-associated pneumonia. *Int J Antimicrob Agents* 2022; **59**: 106487. <https://doi.org/10.1016/j.ijantimicag.2021.106487>

95. Furuie H, Saisho Y, Yoshikawa T*, et al.* Intrapulmonary pharmacokinetics of S-013420, a novel bicyclolide antibacterial, in healthy Japanese subjects. *Antimicrob Agents Chemother* 2010; **54**: 866-70. <https://doi.org/10.1128/AAC.00567-09>

96. Furuie H, Tanioka S, Shimizu K*, et al.* Intrapulmonary pharmacokinetics of lascufloxacin in healthy adult volunteers. *Antimicrob Agents Chemother* 2018; **62**: e02169-17. <https://doi.org/10.1128/AAC.02169-17>

97. Gotfried MH, Danziger LH, Rodvold KA. Steady-state plasma and intrapulmonary concentrations of levofloxacin and ciprofloxacin in healthy adult subjects. *Chest* 2001; **119**: 1114-22. <https://doi.org/10.1378/chest.119.4.1114>

98. Gotfried MH, Danziger LH, Rodvold KA. Steady-state plasma and bronchopulmonary characteristics of clarithromycin extended-release tablets in normal healthy adult subjects. *J Antimicrob Chemother* 2003; **52**: 450-6. <https://doi.org/10.1093/jac/dkg355>

99. Gotfried MH, Horn K, Garrity-Ryan L*, et al.* Comparison of Omadacycline and Tigecycline Pharmacokinetics in the Plasma, Epithelial Lining Fluid, and Alveolar Cells of Healthy Adult Subjects. *Antimicrob Agents Chemother* 2017; **61**: e01135-17. <https://doi.org/10.1128/aac.01135-17>

100. Housman ST, Pope JS, Russomanno J*, et al.* Pulmonary disposition of tedizolid following administration of once-daily oral 200-milligram tedizolid phosphate in healthy adult volunteers. *Antimicrob Agents Chemother* 2012; **56**: 2627-34. <https://doi.org/10.1128/AAC.05354-11>

101. Huang H, Wang Y, Jiang C*, et al.* Intrapulmonary concentration of levofloxacin in patients with idiopathic pulmonary fibrosis. *Pulm Pharmacol Ther* 2014; **28**: 49-52. <https://doi.org/10.1016/j.pupt.2013.10.004>

102. Husain S, Capitano B, Corcoran T*, et al.* Intrapulmonary disposition of amphotericin B after aerosolized delivery of amphotericin B lipid complex (Abelcet; ABLC) in lung transplant recipients. *Transplantation* 2010; **90**: 1215-9. <https://doi.org/10.1097/TP.0b013e3181f995ea>

103. Imberti R, Cusato M, Villani P*, et al.* Steady-state pharmacokinetics and BAL concentration of colistin in critically Ill patients after IV colistin methanesulfonate administration. *Chest* 2010; **138**: 1333-9. <https://doi.org/10.1378/chest.10-0463>

104. Kadota JI, Ishimatsu Y, Iwashita T*, et al.* Intrapulmonary pharmacokinetics of telithromycin, a new ketolide, in healthy Japanese volunteers. *Antimicrob Agents Chemother* 2002; **46**: 917-21. <https://doi.org/10.1128/AAC.46.3.917-921.2002>

105. Katsube T, Saisho Y, Shimada J*, et al.* Intrapulmonary pharmacokinetics of cefiderocol, a novel siderophore cephalosporin, in healthy adult subjects. *J Antimicrob Chemother* 2019; **74**: 1971-4. <https://doi.org/10.1093/jac/dkz123>

106. Katsube T, Nicolau DP, Rodvold KA*, et al.* Intrapulmonary pharmacokinetic profile of cefiderocol in mechanically ventilated patients with pneumonia. *J Antimicrob Chemother* 2021; **76**: 2902-5. <https://doi.org/10.1093/jac/dkab280>

107. Lamer C, de Beco V, Soler P*, et al.* Analysis of vancomycin entry into pulmonary lining fluid by bronchoalveolar lavage in critically ill patients. *Antimicrob Agents Chemother* 1993; **37**: 281-6. <https://doi.org/10.1128/aac.37.2.281>

108. Lodise Jr TP, Gotfried M, Barriere S*, et al.* Telavancin penetration into human epithelial lining fluid determined by population pharmacokinetic modeling and Monte Carlo simulation. *Antimicrob Agents Chemother* 2008; **52**: 2300-4. <https://doi.org/10.1128/AAC.01110-07>

109. Lodise TP, Kinzig-Schippers M, Drusano GL*, et al.* Use of population pharmacokinetic modeling and Monte Carlo simulation to describe the pharmacodynamic profile of cefditoren in plasma and epithelial lining fluid. *Antimicrob Agents Chemother* 2008; **52**: 1945-51. <https://doi.org/10.1128/AAC.00736-06>

110. Lodise TP, Drusano GL, Butterfield JM*, et al.* Penetration of vancomycin into epithelial lining fluid in healthy volunteers. *Antimicrob Agents Chemother* 2011; **55**: 5507-11. <https://doi.org/10.1128/aac.00712-11>

111. Lodise TP, Sorgel F, Melnick D*, et al.* Penetration of meropenem into epithelial lining fluid of patients with ventilator-associated pneumonia. *Antimicrob Agents Chemother* 2011; **55**: 1606-10. <https://doi.org/10.1128/aac.01330-10>

112. Luyt CE, Eldon MA, Stass H*, et al.* Pharmacokinetics and tolerability of amikacin administered as BAY41-6551 aerosol in mechanically ventilated patients with gram-negative pneumonia and acute renal failure. *J Aerosol Med Pulm Drug Deliv* 2011; **24**: 183-90. <https://doi.org/10.1089/jamp.2010.0860>

113. McCallum AD, Pertinez HE, Else LJ*, et al.* Intrapulmonary Pharmacokinetics of First-line Anti-tuberculosis Drugs in Malawian Patients with Tuberculosis. *Clin Infect Dis* 2021; **73**: E3365-E73. <https://doi.org/10.1093/cid/ciaa1265>

114. Mendivil CO, Teeter JG, Finch GL*, et al.* Trough insulin levels in bronchoalveolar lavage following inhaled human insulin (Exubera) in patients with diabetes mellitus. *Diabetes Technology and Therapeutics* 2012; **14**: 50-8. <https://doi.org/10.1089/dia.2011.0148>

115. Muller-Serieys C, Soler P, Cantalloube C*, et al.* Bronchopulmonary disposition of the ketolide telithromycin (HMR 3647). *Antimicrob Agents Chemother* 2001; **45**: 3104-8. <https://doi.org/10.1128/aac.45.11.3104-3108.2001>

116. Jaspard M, Butel N, El Helali N*, et al.* Linezolid-Associated Neurologic Adverse Events in Patients with Multidrug-Resistant Tuberculosis, France. *Emerg Infect Dis* 2020; **26**: 1792-800. <https://doi.org/10.3201/eid2608.191499>

117. Nicasio AM, Tessier PR, Nicolau DP*, et al.* Bronchopulmonary disposition of micafungin in healthy adult volunteers. *Antimicrob Agents Chemother* 2009; **53**: 1218-20. <https://doi.org/10.1128/aac.01386-08>

118. Nicolau DP, Sutherland C, Winget D*, et al.* Bronchopulmonary pharmacokinetic and pharmacodynamic profiles of levofloxacin 750mg once daily in adults undergoing treatment for acute exacerbation of chronic bronchitis. *Pulm Pharmacol Ther* 2012; **25**: 94-8. <https://doi.org/10.1016/j.pupt.2011.12.007>

119. Nicolau DP, Siew L, Armstrong J*, et al.* Phase 1 study assessing the steady-state concentration of ceftazidime and avibactam in plasma and epithelial lining fluid following two dosing regimens. *J Antimicrob Chemother* 2015; **70**: 2862-9. <https://doi.org/10.1093/jac/dkv170>

120. O'Brien JK, Doerfler ME, Harkin TJ*, et al.* Isoniazid levels in the bronchoalveolar lavage fluid of patients with pulmonary tuberculosis. *Lung* 1998; **176**: 205-11. <https://doi.org/10.1007/pl00007603>

121. Olsen KM, San Pedro G, Gann LP*, et al.* Intrapulmonary pharmacokinetics of azithromycin in healthy volunteers given five oral doses. *Antimicrob Agents Chemother* 1996; **40**: 2582-5. <https://doi.org/10.1128/aac.40.11.2582>

122. Ong CT, Dandekar PK, Sutherland C*, et al.* Intrapulmonary concentrations of telithromycin: Clinical implications for respiratory tract infections due to Streptococcus pneumoniae. *Chemotherapy* 2005; **51(6)**: 339-46. <https://doi.org/10.1159/000088958>

123. Paiboonvong T, Nosoongnoen W, Sathirakul K*, et al.* Pharmacokinetics and penetration of sitafloxacin into alveolar epithelial lining fluid in critically ill Thai patients with pneumonia. *Antimicrob Agents Chemother* 2019; **63**: e00800-19. <https://doi.org/10.1128/AAC.00800-19>

124. Panidis D, Markantonis SL, Boutzouka E*, et al.* Penetration of gentamicin into the alveolar lining fluid of critically ill patients with ventilator-associated pneumonia. *Chest* 2005; **128**: 545-52. <https://doi.org/10.1378/chest.128.2.545>

125. Patel KB, Xuan D, Tessier PR*, et al.* Comparison of bronchopulmonary pharmacokinetics of clarithromycin and azithromycin. *Antimicrob Agents Chemother* 1996; **40**: 2375-9. <https://doi.org/10.1128/aac.40.10.2375>

126. Riccobene TA, Pushkin R, Jandourek A*, et al.* Penetration of Ceftaroline into the Epithelial Lining Fluid of Healthy Adult Subjects. *Antimicrob Agents Chemother* 2016; **60**: 5849-57. <https://doi.org/10.1128/aac.02755-15>

127. Rizk ML, Rhee EG, Jumes PA*, et al.* Intrapulmonary Pharmacokinetics of Relebactam, a Novel β-Lactamase Inhibitor, Dosed in Combination with Imipenem-Cilastatin in Healthy Subjects. *Antimicrob Agents Chemother* 2018; **62**: e01411-17. <https://doi.org/10.1128/aac.01411-17>

128. Rodvold KA, Gotfried MH, Danziger LH*, et al.* Intrapulmonary steady-state concentrations of clarithromycin and azithromycin in healthy adult volunteers. *Antimicrob Agents Chemother* 1997; **41**: 1399-402. <https://doi.org/10.1128/AAC.41.6.1399>

129. Rodvold KA, Danziger LH, Gotfried MH. Steady-state plasma and bronchopulmonary concentrations of intravenous levofloxacin and azithromycin in healthy adults. *Antimicrob Agents Chemother* 2003; **47**: 2450-7. <https://doi.org/10.1128/aac.47.8.2450-2457.2003>

130. Rodvold KA, Gotfried MH, Still JG*, et al.* Comparison of plasma, epithelial lining fluid, and alveolar macrophage concentrations of solithromycin (CEM-101) in healthy adult subjects. *Antimicrob Agents Chemother* 2012; **56**: 5076-81. <https://doi.org/10.1128/AAC.00766-12>

131. Rodvold KA, Gotfried MH, Chugh R*, et al.* Comparison of plasma and intrapulmonary concentrations of nafithromycin (WCK 4873) in healthy adult subjects. *Antimicrob Agents Chemother* 2017; **61**: e01096-17. <https://doi.org/10.1128/AAC.01096-17>

132. Rodvold KA, Gotfried MH, Chugh R*, et al.* Plasma and intrapulmonary concentrations of cefepime and zidebactam following intravenous administration of wck 5222 to healthy adult subjects. *Antimicrob Agents Chemother* 2018; **62**: e00682-18. <https://doi.org/10.1128/AAC.00682-18>

133. Rodvold KA, Gotfried MH, Chugh R*, et al.* Intrapulmonary pharmacokinetics of levonadifloxacin following oral administration of alalevonadifloxacin to healthy adult subjects. *Antimicrob Agents Chemother* 2018; **62**: e02297-17. <https://doi.org/10.1128/AAC.02297-17>

134. Rodvold KA, Bader J, Bruss JB*, et al.* Pharmacokinetics of SPR206 in Plasma, Pulmonary Epithelial Lining Fluid, and Alveolar Macrophages following Intravenous Administration to Healthy Adult Subjects. *Antimicrob Agents Chemother* 2023; **67**: e0042623. <https://doi.org/10.1128/aac.00426-23>

135. Shelton MJ, Lovern M, Ng-Cashin J*, et al.* Zanamivir pharmacokinetics and pulmonary penetration into epithelial lining fluid following intravenous or oral inhaled administration to healthy adult subjects. *Antimicrob Agents Chemother* 2011; **55**: 5178-84. <https://doi.org/10.1128/AAC.00703-11>

136. Tayman C, El-Attug MN, Adams E*, et al.* Quantification of amikacin in bronchial epithelial lining fluid in neonates. *Antimicrob Agents Chemother* 2011; **55**: 3990-3. <https://doi.org/10.1128/aac.00277-11>

137. Tenero D, Bowers G, Rodvold KA*, et al.* Intrapulmonary pharmacokinetics of GSK2251052 in healthy volunteers. *Antimicrob Agents Chemother* 2013; **57**: 3334-9. <https://doi.org/10.1128/AAC.02483-12>

138. Twigg HL, Schnizlein-Bick CT, Weiden M*, et al.* Measurement of antiretroviral drugs in the lungs of HIV-infected patients. *HIV Ther* 2010; **4**: 247-51. <https://doi.org/10.2217/hiv.10.5>

139. Walsh TJ, Goutelle S, Jelliffe RW*, et al.* Intrapulmonary pharmacokinetics and pharmacodynamics of micafungin in adult lung transplant patients. *Antimicrob Agents Chemother* 2010; **54**: 3451-9. <https://doi.org/10.1128/AAC.01647-09>

140. Wenzler E, Gotfried MH, Loutit JS*, et al.* Meropenem-RPX7009 Concentrations in Plasma, Epithelial Lining Fluid, and Alveolar Macrophages of Healthy Adult Subjects. *Antimicrob Agents Chemother* 2015; **59**: 7232-9. <https://doi.org/10.1128/aac.01713-15>

141. Zeitlinger M, Schwameis R, Burian A*, et al.* Simultaneous assessment of the pharmacokinetics of a pleuromutilin, lefamulin, in plasma, soft tissues and pulmonary epithelial lining fluid. *J Antimicrob Chemother* 2016; **71**: 1022-6. <https://doi.org/10.1093/jac/dkv442>

142. Zhang J, Xie X, Zhou X*, et al.* Permeability and concentration of levofloxacin in epithelial lining fluid in patients with lower respiratory tract infections. *J Clin Pharmacol* 2010; **50**: 922-8. <https://doi.org/10.1177/0091270009355160>

143. Athanassa ZE, Markantonis SL, Fousteri MZF*, et al.* Pharmacokinetics of inhaled colistimethate sodium (CMS) in mechanically ventilated critically ill patients. *Intensive Care Med* 2012; **38**: 1779-86. <https://doi.org/10.1007/s00134-012-2628-7>

144. Boisson M, Jacobs M, Grégoire N*, et al.* Comparison of intrapulmonary and systemic pharmacokinetics of colistin methanesulfonate (CMS) and colistin after aerosol delivery and intravenous administration of CMS in critically ill patients. *Antimicrob Agents Chemother* 2014; **58**: 7331-9. <https://doi.org/10.1128/AAC.03510-14>

145. Boselli E, Breilh D, Duflo F*, et al.* Steady-state plasma and intrapulmonary concentrations of cefepime administered in continuous infusion in critically ill patients with severe nosocomial pneumonia. *Crit Care Med* 2003; **31**: 2102-6. <https://doi.org/10.1097/01.Ccm.0000069734.38738.C8>

146. Boselli E, Breilh D, Cannesson M*, et al.* Steady-state plasma and intrapulmonary concentrations of piperacillin/tazobactam 4 g/0.5 g administered to critically ill patients with severe nosocomial pneumonia. *Intensive Care Med* 2004; **30**: 976-9. <https://doi.org/10.1007/s00134-004-2222-8>

147. Boselli E, Breilh D, Rimmelé T*, et al.* Plasma and lung concentrations of ceftazidime administered in continuous infusion to critically ill patients with severe nosocomial pneumonia. *Intensive Care Med* 2004; **30**: 989-91. <https://doi.org/10.1007/s00134-004-2171-2>

148. Boselli E, Breilh D, Rimmelé T*, et al.* Pharmacokinetics and intrapulmonary diffusion of levofloxacin in critically ill patients with severe community-acquired pneumonia. *Crit Care Med* 2005; **33**: 104-9. <https://doi.org/10.1097/01.ccm.0000150265.42067.4c>

149. Boselli E, Breilh D, Rimmelé T*, et al.* Pharmacokinetics and intrapulmonary concentrations of linezolid administered to critically ill patients with ventilator-associated pneumonia. *Crit Care Med* 2005; **33**: 1529-33. <https://doi.org/10.1097/01.ccm.0000168206.59873.80>

150. Boselli E, Breilh D, Saux MC*, et al.* Pharmacokinetics and lung concentrations of ertapenem in patients with ventilator-associated pneumonia. *Intensive Care Med* 2006; **32**: 2059-62. <https://doi.org/10.1007/s00134-006-0401-5>

151. Boselli E, Breilh D, Djabarouti S*, et al.* Reliability of mini-bronchoalveolar lavage for the measurement of epithelial lining fluid concentrations of tobramycin in critically ill patients. *Intensive Care Med* 2007; **33**: 1519-23. <https://doi.org/10.1007/s00134-007-0688-x>

152. De Pascale G, Lisi L, Ciotti GMP*, et al.* Pharmacokinetics of high-dose tigecycline in critically ill patients with severe infections. *Annals of Intensive Care* 2020; **10**: 94. <https://doi.org/10.1186/s13613-020-00715-2>

153. Felton TW, McCalman K, Malagon I*, et al.* Pulmonary Penetration of Piperacillin and Tazobactam in Critically Ill Patients. *Clin Pharmacol Ther* 2014; **96**: 438-48. <https://doi.org/10.1038/clpt.2014.131>

154. Gkoufa A, Sou T, Karaiskos I*, et al.* Pulmonary and systemic pharmacokinetics of colistin methanesulfonate (CMS) and formed colistin following nebulisation of CMS among patients with ventilator-associated pneumonia. *Int J Antimicrob Agents* 2022; **59**: 106588. <https://doi.org/10.1016/j.ijantimicag.2022.106588>

155. Layios N, Visée C, Mistretta V*, et al.* Modelled Target Attainment after Temocillin Treatment in Severe Pneumonia: Systemic and Epithelial Lining Fluid Pharmacokinetics of Continuous versus Intermittent Infusions. *Antimicrob Agents Chemother* 2022; **66**: e0205221. <https://doi.org/10.1128/aac.02052-21>

156. Toutain J, Boselli E, Djabarouti S*, et al.* Determination of linezolid in plasma and bronchoalveolar lavage by high-performance liquid chromatography with ultraviolet detection using a fully automated extraction method. *J Chromatogr B Analyt Technol Biomed Life Sci* 2004; **813**: 145-50. <https://doi.org/10.1016/j.jchromb.2004.09.030>

157. Wu C, Zhang X, Xie J*, et al.* Pharmacokinetic/Pharmacodynamic Parameters of Linezolid in the Epithelial Lining Fluid of Patients With Sepsis. *J Clin Pharmacol* 2022; **62**: 891-7. <https://doi.org/https://dx.doi.org/10.1002/jcph.2031>

158. Funatsu Y, Hasegawa N, Fujiwara H*, et al.* Pharmacokinetics of arbekacin in bronchial epithelial lining fluid of healthy volunteers. *J Infect Chemother* 2014; **20**: 607-11. <https://doi.org/10.1016/j.jiac.2014.05.007>

159. Funatsu Y, Tasaka S, Asami T*, et al.* Pharmacokinetics of intravenous peramivir in the airway epithelial lining fluid of healthy volunteers. *Antivir Ther* 2016; **21**: 621-5. <https://doi.org/10.3851/IMP3096>

160. Hasegawa N, Nishimura T, Watabnabe M*, et al.* Concentrations of clarithromycin and active metabolite in the epithelial lining fluid of patients with Mycobacterium avium complex pulmonary disease. *Pulm Pharmacol Ther* 2009; **22**: 190-3. <https://doi.org/10.1016/j.pupt.2008.11.004>

161. Kikuchi J, Yamazaki K, Kikuchi E*, et al.* Pharmacokinetics of gatifloxacin after a single oral dose in healthy young adult subjects and adult patients with chronic bronchitis, with a comparison of drug concentrations obtained by bronchoscopic microsampling and bronchoalveolar lavage. *Clin Ther* 2007; **29**: 123-30. <https://doi.org/10.1016/j.clinthera.2007.01.005>

162. Kikuchi J, Yamazaki K, Kikuchi E*, et al.* Pharmacokinetics of telithromycin using bronchoscopic microsampling after single and multiple oral doses. *Pulm Pharmacol Ther* 2007; **20**: 549-55. <https://doi.org/10.1016/j.pupt.2006.05.006>

163. Kikuchi E, Yamazaki K, Kikuchi J*, et al.* Pharmacokinetics of clarithromycin in bronchial epithelial lining fluid. *Respirology* 2008; **13**: 221-6. <https://doi.org/10.1111/j.1440-1843.2007.01208.x>

164. Kikuchi E, Kikuchi J, Nasuhara Y*, et al.* Comparison of the pharmacodynamics of biapenem in bronchial epithelial lining fluid in healthy volunteers given half-hour and three-hour intravenous infusions. *Antimicrob Agents Chemother* 2009; **53**: 2799-803. <https://doi.org/10.1128/AAC.01578-08>

165. Yamazaki K, Ogura S, Ishizaka A*, et al.* Bronchoscopic microsampling method for measuring drug concentration in epithelial lining fluid. *Am J Respir Crit Care Med* 2003; **168**: 1304-7. <https://doi.org/10.1164/rccm.200301-111OC>

166. Bergogne-Berezin E, Muller-Serieys C, Kafe H. Penetration of lomefloxacin into bronchial secretions following single and multiple oral administration. *Am J Med* 1992; **92**: S8-S11. <https://doi.org/10.1016/0002-9343(92)90299-q>

167. Bergogne-Berezin E, Muller-Serieys C, Aubier M*, et al.* Concentration of meropenem in serum and in bronchial secretions in patients undergoing fibreoptic bronchoscopy. *Eur J Clin Pharmacol* 1994; **46**: 87-8. <https://doi.org/10.1007/BF00195922>

168. Jehl F, Muller-Serieys C, de Larminat V*, et al.* Penetration of piperacillin-tazobactam into bronchial secretions after multiple doses to intensive care patients. *Antimicrob Agents Chemother* 1994; **38**: 2780-4. <https://doi.org/10.1128/aac.38.12.2780>

169. Kontou P, Chatzika K, Pitsiou G*, et al.* Pharmacokinetics of ciprofloxacin and its penetration into bronchial secretions of mechanically ventilated patients with chronic obstructive pulmonary disease. *Antimicrob Agents Chemother* 2011; **55**: 4149-53. <https://doi.org/10.1128/AAC.00566-10>

170. Leone M, Albanese J, Sampol-Manos E*, et al.* Moxifloxacin Penetration in Bronchial Secretions of Mechanically Ventilated Patients with Pneumonia. *Antimicrob Agents Chemother* 2004; **48**: 638-40. <https://doi.org/10.1128/AAC.48.2.638-640.2004>

171. Monforte V, Roman A, Gavalda J*, et al.* Nebulized amphotericin B concentration and distribution in the respiratory tract of lung-transplanted patients. *Transplantation* 2003; **75**: 1571-4. <https://doi.org/10.1097/01.TP.0000054233.60100.7A>

172. Rebholz D, Liebchen U, Paal M*, et al.* Can linezolid be validly measured in endotracheal aspiration in critically ill patients? A proof-of-concept trial. *Intensive Care Med Exp* 2024; **12**: 47. <https://doi.org/10.1186/s40635-024-00630-x>

173. Santré C, Georges H, Jacquier JM*, et al.* Amikacin levels in bronchial secretions of 10 pneumonia patients with respiratory support treated once daily versus twice daily. *Antimicrob Agents Chemother* 1995; **39**: 264-7. <https://doi.org/10.1128/aac.39.1.264>

174. Saux P, Martin C, Mallet MN*, et al.* Penetration of ciprofloxacin into bronchial secretions from mechanically ventilated patients with nosocomial bronchopneumonia. *Antimicrob Agents Chemother* 1994; **38**: 901-4. <https://doi.org/10.1128/aac.38.4.901>

175. Simon N, Sampol E, Albanese J*, et al.* Population pharmacokinetics of moxifloxacin in plasma and bronchial secretions in patients with severe bronchopneumonia. *Clin Pharmacol Ther* 2003; **74**: 353-63. <https://doi.org/10.1016/S0009-9236(03)00201-7>

176. Valcke YJ, Vogelaers DP, Colardyn FA*, et al.* Penetration of netilmicin in the lower respiratory tract after once-daily dosing. *Chest* 1992; **101**: 1028-32. <https://doi.org/10.1378/chest.101.4.1028>

177. Edlinger-Stanger M, Al Jalali V, Andreas M*, et al.* Plasma and Lung Tissue Pharmacokinetics of Ceftaroline Fosamil in Patients Undergoing Cardiac Surgery with Cardiopulmonary Bypass: an In Vivo Microdialysis Study. *Antimicrob Agents Chemother* 2021; **65**: e0067921. <https://doi.org/10.1128/AAC.00679-21>

178. Herkner H, Muller MR, Kreischitz N*, et al.* Closed-chest microdialysis to measure antibiotic penetration into human lung tissue. *Am J Respir Crit Care Med* 2002; **165**: 273-6. <https://doi.org/10.1164/ajrccm.165.2.2106082>

179. Hutschala D, Skhirtladze K, Zuckermann A*, et al.* In vivo measurement of levofloxacin penetration into lung tissue after cardiac surgery. *Antimicrob Agents Chemother* 2005; **49**: 5107-11. <https://doi.org/10.1128/AAC.49.12.5107-5111.2005>

180. Hutschala D, Kinstner C, Skhirtladze K*, et al.* The impact of perioperative atelectasis on antibiotic penetration into lung tissue: An in vivo microdialysis study. *Intensive Care Med* 2008; **34**: 1827-34. <https://doi.org/10.1007/s00134-008-1122-8>

181. Lindenmann J, Kugler SA, Matzi V*, et al.* High extracellular levels of cefpirome in unaffected and infected lung tissue of patients. *J Antimicrob Chemother* 2011; **66**: 160-4. <https://doi.org/10.1093/jac/dkq413>

182. Matzi V, Lindenmann J, Porubsky C*, et al.* Extracellular concentrations of fosfomycin in lung tissue of septic patients. *J Antimicrob Chemother* 2010; **65**: 995-8. <https://doi.org/10.1093/jac/dkq070>

183. Tomaselli F, Dittrich P, Maier A*, et al.* Penetration of piperacillin and tazobactam into pneumonic human lung tissue measured by in vivo microdialysis. *Br J Clin Pharmacol* 2003; **55**: 620-4. <https://doi.org/10.1046/j.1365-2125.2003.01797.x>

184. Tomaselli F, Maier A, Matzi V*, et al.* Penetration of meropenem into pneunomic human lung tissue as measured by in vivo microdialysis. *Antimicrob Agents Chemother* 2004; **48**: 2228-32. <https://doi.org/10.1128/AAC.48.6.2228-2232.2004>

185. Zeitlinger MA, Traunmüller F, Abrahim A*, et al.* A pilot study testing whether concentrations of levofloxacin in interstitial space fluid of soft tissues may serve as a surrogate for predicting its pharmacokinetics in lung. *Int J Antimicrob Agents* 2007; **29**: 44-50. <https://doi.org/10.1016/j.ijantimicag.2006.08.045>

186. Heinrichs MT, Vashakidze S, Nikolaishvili K*, et al.* Moxifloxacin target site concentrations in patients with pulmonary TB utilizing microdialysis: A clinical pharmacokinetic study. *J Antimicrob Chemother* 2018; **73**: 477-83. <https://doi.org/10.1093/jac/dkx421>

187. Kempker RR, Barth AB, Vashakidze S*, et al.* Cavitary penetration of levofloxacin among patients with multidrug-resistant tuberculosis. *Antimicrob Agents Chemother* 2015; **59**: 3149-55. <https://doi.org/10.1128/AAC.00379-15>

188. Kempker RR, Heinrichs MT, Nikolaishvili K*, et al.* Lung tissue concentrations of pyrazinamide among patients with drug-resistant pulmonary tuberculosis. *Antimicrob Agents Chemother* 2017; **61**: e00226-17. <https://doi.org/10.1128/AAC.00226-17>

189. Brunner M, Langer O, Dobrozemsky G*, et al.* [18F]Ciprofloxacin, a new positron emission tomography tracer for noninvasive assessment of the tissue distribution and pharmacokinetics of ciprofloxacin in humans. *Antimicrob Agents Chemother* 2004; **48**: 3850-7. <https://doi.org/10.1128/aac.48.10.3850-3857.2004>

190. Fischman AJ, Alpert NM, Livni E*, et al.* Pharmacokinetics of 18F-labeled fluconazole in healthy human subjects by positron emission tomography. *Antimicrob Agents Chemother* 1993; **37**: 1270-7. <https://doi.org/10.1128/aac.37.6.1270>

191. Fischman AJ, Livni E, Babich J*, et al.* Pharmacokinetics of [18F]fleroxacin in healthy human subjects studied by using positron emission tomography. *Antimicrob Agents Chemother* 1993; **37**: 2144-52. <https://doi.org/10.1128/aac.37.10.2144>

192. Fischman AJ, Livni E, Babich JW*, et al.* Pharmacokinetics of [18F]fleroxacin in patients with acute exacerbations of chronic bronchitis and complicated urinary tract infection studied by positron emission tomography. *Antimicrob Agents Chemother* 1996; **40**: 659-64. <https://doi.org/10.1128/aac.40.3.659>

193. Fischman AJ, Babich JW, Bonab AA*, et al.* Pharmacokinetics of [18F]trovafloxacin in healthy human subjects studied with positron emission tomography. *Antimicrob Agents Chemother* 1998; **42**: 2048-54. <https://doi.org/10.1128/aac.42.8.2048>

194. Garg PK, Lokitz SJ, Nazih R*, et al.* Biodistribution and radiation dosimetry of 11c-nicotine from whole-body PET imaging in humans. *J Nucl Med* 2017; **58**: 473-8. <https://doi.org/10.2967/jnumed.116.180059>

195. Harris SM, Davis JC, Snyder SE*, et al.* Evaluation of the biodistribution of 11C-methionine in children and young adults. *J Nucl Med* 2013; **54**: 1902-8. <https://doi.org/10.2967/jnumed.112.118125>

196. Ordonez AA, Wang H, Magombedze G*, et al.* Dynamic imaging in patients with tuberculosis reveals heterogeneous drug exposures in pulmonary lesions. *Nat Med* 2020; **26**: 529-34. <https://doi.org/10.1038/s41591-020-0770-2>

197. Van Der Veldt AAM, Lubberink M, Greuter HN*, et al.* Absolute Quantification of [(11)C]docetaxel kinetics in lung cancer patients using positron emission tomography. *Clin Cancer Res* 2011; **17**: 4814-24. <https://doi.org/10.1158/1078-0432.CCR-10-2933>

198. Van Der Veldt AAM, Lubberink M, Mathijssen RHJ*, et al.* Toward prediction of efficacy of chemotherapy: a proof of concept study in lung cancer patients using [(11)C]docetaxel and positron emission tomography. *Clin Cancer Res* 2013; **19**: 4163-73. <https://doi.org/10.1158/1078-0432.CCR-12-3779>

199. Volkow ND, Fowler JS, Wolf AP*, et al.* Distribution and kinetics of carbon-11-cocaine in the human body measured with PET. *J Nucl Med* 1992; **33**: 521-5.

200. Volkow ND, Fowler JS, Wang GJ*, et al.* Distribution and pharmacokinetics of methamphetamine in the human body: Clinical implications. *PLoS One* 2010; **5**: e15269. <https://doi.org/10.1371/journal.pone.0015269>

201. Cicchese JM, Dartois V, Kirschner DE*, et al.* Both Pharmacokinetic Variability and Granuloma Heterogeneity Impact the Ability of the First-Line Antibioticsw to Sterilize Tuberculosis Granulomas. *Front Pharmacol* 2020; **11**: 333. <https://doi.org/10.3389/fphar.2020.00333>

202. Clewe O, Goutelle S, Conte JE*, et al.* A pharmacometric pulmonary model predicting the extent and rate of distribution from plasma to epithelial lining fluid and alveolar cells - Using rifampicin as an example. *Eur J Clin Pharmacol* 2015; **71**: 313-9. <https://doi.org/10.1007/s00228-014-1798-3>

203. Drusano GL, Preston SL, Gotfried MH*, et al.* Levofloxacin penetration into epithelial lining fluid as determined by population pharmacokinetic modeling and Monte Carlo simulation. *Antimicrob Agents Chemother* 2002; **46**: 586-9. <https://doi.org/10.1128/AAC.46.2.586-589.2002>

204. Drusano GL, Lodise TP, Melnick D*, et al.* Meropenem penetration into epithelial lining fluid in mice and humans and delineation of exposure targets. *Antimicrob Agents Chemother* 2011; **55**: 3406-12. <https://doi.org/10.1128/aac.01559-10>

205. Felton TW, Ogungbenro K, Boselli E*, et al.* Comparison of piperacillin exposure in the lungs of critically ill patients and healthy volunteers. *J Antimicrob Chemother* 2018; **73**: 1340-7. <https://doi.org/10.1093/jac/dkx541>

206. Goutelle S, Bourguignon L, Maire PH*, et al.* Population modeling and Monte Carlo simulation study of the pharmacokinetics and antituberculosis pharmacodynamics of rifampin in lungs. *Antimicrob Agents Chemother* 2009; **53**: 2974-81. <https://doi.org/10.1128/aac.01520-08>

207. Hughes JH, Sweeney K, Ahadieh S*, et al.* Predictions of Systemic, Intracellular, and Lung Concentrations of Azithromycin With Different Dosing Regimens Used in COVID-19 Clinical Trials. *CPT Pharmacometrics Syst Pharmacol* 2020; **9**: 435-43. <https://doi.org/10.1002/psp4.12537>

208. Ikawa K, Kikuchi E, Kikuchi J*, et al.* Pharmacokinetic modelling of serum and bronchial concentrations for clarithromycin and telithromycin, and site-specific pharmacodynamic simulation for their dosages. *J Clin Pharm Ther* 2014; **39**: 411-7. <https://doi.org/10.1111/jcpt.12157>

209. Kawaguchi N, Katsube T, Echols R*, et al.* Intrapulmonary Pharmacokinetic Modeling and Simulation of Cefiderocol, a Parenteral Siderophore Cephalosporin, in Patients With Pneumonia and Healthy Subjects. *J Clin Pharmacol* 2022; **62**: 670-80. <https://doi.org/10.1002/jcph.1986>

210. Kuti JL, Nicolau DP. Presence of infection influences the epithelial lining fluid penetration of oral levofloxacin in adult patients. *Int J Antimicrob Agents* 2015; **45**: 512-8. <https://doi.org/10.1016/j.ijantimicag.2014.12.028>

211. Rubino CM, Ma L, Bhavnani SM*, et al.* Evaluation of tigecycline penetration into colon wall tissue and epithelial lining fluid using a population pharmacokinetic model and Monte Carlo simulation. *Antimicrob Agents Chemother* 2007; **51**: 4085-9. <https://doi.org/10.1128/aac.00065-07>

212. Shorr AF, Bruno CJ, Zhang Z*, et al.* Ceftolozane/tazobactam probability of target attainment and outcomes in participants with augmented renal clearance from the randomized phase 3 ASPECT-NP trial. *Critical Care* 2021; **25**: 354. <https://doi.org/10.1186/s13054-021-03773-5>

213. Van Hasselt JGC, Rizk ML, Lala M*, et al.* Pooled population pharmacokinetic model of imipenem in plasma and the lung epithelial lining fluid. *Br J Clin Pharmacol* 2016; **81**: 1113-23. <https://doi.org/10.1111/bcp.12901>

214. Xiao AJ, Miller BW, Huntington JA*, et al.* Ceftolozane/tazobactam pharmacokinetic/pharmacodynamic-derived dose justification for phase 3 studies in patients with nosocomial pneumonia. *J Clin Pharmacol* 2016; **56**: 56-66. <https://doi.org/10.1002/jcph.566>

215. Zimmerman M, Lestner J, Prideaux B*, et al.* Ethambutol Partitioning in Tuberculous Pulmonary Lesions Explains Its Clinical Efficacy. *Antimicrob Agents Chemother* 2017; **61**: e00924-17. <https://doi.org/10.1128/aac.00924-17>

216. Zhang Z, Patel YT, Fiedler-Kelly J*, et al.* Population Pharmacokinetic Analysis for Plasma and Epithelial Lining Fluid Ceftolozane/Tazobactam Concentrations in Patients With Ventilated Nosocomial Pneumonia. *J Clin Pharmacol* 2021; **61**: 254-68. <https://doi.org/10.1002/jcph.1733>

217. An G, Morris ME. A physiologically based pharmacokinetic model of mitoxantrone in mice and scale-up to humans: A semi-mechanistic model incorporating DNA and protein binding. *AAPS Journal* 2012; **14**: 352-64. <https://doi.org/10.1208/s12248-012-9344-7>

218. Aulin LBS, Tandar ST, van Zijp T*, et al.* Physiologically Based Modelling Framework for Prediction of Pulmonary Pharmacokinetics of Antimicrobial Target Site Concentrations. *Clin Pharmacokinet* 2022; **61**: 1735-48. <https://doi.org/10.1007/s40262-022-01186-3>

219. Bae DJ, Kim SY, Bae SM*, et al.* Whole-Body Physiologically Based Pharmacokinetic Modeling of Trastuzumab and Prediction of Human Pharmacokinetics. *J Pharm Sci* 2019; **108**: 2180-90. <https://doi.org/10.1016/j.xphs.2019.01.024>

220. Chao FC, Manaia EB, Ponchel G*, et al.* A physiologically-based pharmacokinetic model for predicting doxorubicin disposition in multiple tissue levels and quantitative toxicity assessment. *Biomed Pharmacother* 2023; **168**: 115636. <https://doi.org/10.1016/j.biopha.2023.115636>

221. Cui C, Zhang M, Yao X*, et al.* Dose selection of chloroquine phosphate for treatment of COVID-19 based on a physiologically based pharmacokinetic model. *Acta Pharmaceutica Sinica B* 2020; **10**: 1216-27. <https://doi.org/10.1016/j.apsb.2020.04.007>

222. Fan J, Yang Y, Grimstein M*, et al.* Whole Body PBPK Modeling of Remdesivir and Its Metabolites to Aid in Estimating Active Metabolite Exposure in the Lung and Liver in Patients With Organ Dysfunction. *Clin Pharmacol Ther* 2022; **111**: 624-34. <https://doi.org/10.1002/cpt.2445>

223. Fu Q, Sun X, Lustburg MB*, et al.* Predicting Paclitaxel Disposition in Humans With Whole-Body Physiologically-Based Pharmacokinetic Modeling. *CPT: Pharmacometrics Syst Pharmacol* 2019; **8**: 931-9. <https://doi.org/10.1002/psp4.12472>

224. Gao G, Law F, Wong RNS*, et al.* A physiologically-based pharmacokinetic model of oseltamivir phosphate and its carboxylate metabolite for rats and humans. *ADMET and DMPK* 2019; **7**: 22-43. <https://doi.org/10.5599/admet.628>

225. Gaohua L, Wedagedera J, Small BG*, et al.* Development of a Multicompartment Permeability-Limited Lung PBPK Model and Its Application in Predicting Pulmonary Pharmacokinetics of Antituberculosis Drugs. *CPT Pharmacometrics Syst Pharmacol* 2015; **4**: 605-13. <https://doi.org/10.1002/psp4.12034>

226. Humphries H, Almond L, Berg A*, et al.* Development of physiologically-based pharmacokinetic models for standard of care and newer tuberculosis drugs. *CPT Pharmacometrics Syst Pharmacol* 2021; **10**: 1382-95. <https://doi.org/10.1002/psp4.12707>

227. Jagdale P, Sepp A, Shah DK. Physiologically-based pharmacokinetic model for pulmonary disposition of protein therapeutics in humans. *J Pharmacokinet Pharmacodyn* 2022; **49**: 607-24. <https://doi.org/10.1007/s10928-022-09824-w>

228. Jermain B, Hanafin PO, Cao Y*, et al.* Development of a Minimal Physiologically-Based Pharmacokinetic Model to Simulate Lung Exposure in Humans Following Oral Administration of Ivermectin for COVID-19 Drug Repurposing. *J Pharm Sci* 2020; **109**: 3574-8. <https://doi.org/10.1016/j.xphs.2020.08.024>

229. Karakitsios E, Dokoumetzidis A. Extrapolation of lung pharmacokinetics of antitubercular drugs from preclinical species to humans using PBPK modelling. *J Antimicrob Chemother* 2024; **79**: 1362-71. <https://doi.org/10.1093/jac/dkae109>

230. Lee JB, Zhou S, Chiang M*, et al.* Interspecies prediction of pharmacokinetics and tissue distribution of doxorubicin by physiologically-based pharmacokinetic modeling. *Biopharm Drug Dispos* 2020; **41**: 192-205. <https://doi.org/10.1002/bdd.2229>

231. Liu X, Jusko WJ. Physiologically based pharmacokinetics of lysosomotropic chloroquine in Rat and Human. *J Pharmacol Exp Ther* 2021; **376**: 261-72. <https://doi.org/10.1124/jpet.120.000385>

232. Martins FS, Martins JES, Severino P*, et al.* Physiologically based pharmacokinetic modelling to inform combination dosing regimens of ceftaroline and daptomycin in special populations. *Br J Clin Pharmacol* 2023; **89**: 2726-38. <https://doi.org/10.1111/bcp.15731>

233. Mehta K, Guo T, van der Graaf PH*, et al.* Predictions of Bedaquiline and Pretomanid Target Attainment in Lung Lesions of Tuberculosis Patients using Translational Minimal Physiologically Based Pharmacokinetic Modeling. *Clin Pharmacokinet* 2023; **62**: 519-32. <https://doi.org/10.1007/s40262-023-01217-7>

234. Mehta K, Balazki P, van der Graaf PH*, et al.* Predictions of Bedaquiline Central Nervous System Exposure in Patients with Tuberculosis Meningitis Using Physiologically based Pharmacokinetic Modeling. *Clin Pharmacokinet* 2024; **63**: 657-68. <https://doi.org/10.1007/s40262-024-01363-6>

235. Muliaditan M, Teutonico D, Ortega-Muro F*, et al.* Prediction of lung exposure to anti-tubercular drugs using plasma pharmacokinetic data: Implications for dose selection. *Eur J Pharm Sci* 2022; **173** 106163. <https://doi.org/10.1016/j.ejps.2022.106163>

236. Rowland Yeo K, Zhang M, Pan X*, et al.* Impact of Disease on Plasma and Lung Exposure of Chloroquine, Hydroxychloroquine and Azithromycin: Application of PBPK Modeling. *Clin Pharmacol Ther* 2020; **108**: 976-84. <https://doi.org/10.1002/cpt.1955>

237. Salerno SN, Edginton A, Cohen-Wolkowiez M*, et al.* Development of an Adult Physiologically Based Pharmacokinetic Model of Solithromycin in Plasma and Epithelial Lining Fluid. *CPT Pharmacometrics Syst Pharmacol* 2017; **6**: 814-22. <https://doi.org/10.1002/psp4.12252>

238. Sharma S, Leonard A, Phoenix K*, et al.* Systemically Administered Anti-uPAR Antibody Plasma and Lung ELF Pharmacokinetics Characterized by Minimal Lung PBPK Model. *AAPS PharmSciTech* 2023; **24**: 236. <https://doi.org/10.1208/s12249-023-02689-3>

239. Shibata M, Masuda M, Sasahara K*, et al.* Prediction of human pharmacokinetic profiles of the antituberculosis drug delamanid from nonclinical data: Potential therapeutic value against extrapulmonary tuberculosis. *Antimicrob Agents Chemother* 2021; **65**: e0257120. <https://doi.org/10.1128/AAC.02571-20>

240. Shin BS, Kim CH, Jun YS*, et al.* Physiologically based pharmacokinetics of bisphenol A. *J Toxicol Environ Health A* 2004; **67**: 1971-85. <https://doi.org/10.1080/15287390490514615>

241. Shin BS, Hong SH, Bulitta JB*, et al.* Physiologically based pharmacokinetics of zearalenone. *J Toxicol Environ Health A* 2009; **72**: 1395-405. <https://doi.org/10.1080/15287390903212741>

242. Shin BS, Bulitta JB, Balthasar JP*, et al.* Prediction of human pharmacokinetics and tissue distribution of apicidin, a potent histone deacetylase inhibitor, by physiologically based pharmacokinetic modeling. *Cancer Chemother Pharmacol* 2011; **68**: 465-75. <https://doi.org/10.1007/s00280-010-1502-y>

243. Sun F, Lee L, Zhang Z*, et al.* Preclinical pharmacokinetic studies of 3-deazaneplanocin A, a potent epigenetic anticancer agent, and its human pharmacokinetic prediction using GastroPlusTM. *Eur J Pharm Sci* 2015; **77**: 290-302. <https://doi.org/10.1016/j.ejps.2015.06.021>

244. Thémans P, Marquet P, Winkin JJ*, et al.* Towards a Generic Tool for Prediction of Meropenem Systemic and Infection-Site Exposure: A Physiologically Based Pharmacokinetic Model for Adult Patients with Pneumonia. *Drugs R D* 2019; **19**: 177-89. <https://doi.org/10.1007/s40268-019-0268-x>

245. Wyska E, Swierczek A, Pociecha K*, et al.* Physiologically based modeling of lisofylline pharmacokinetics following intravenous administration in mice. *Eur J Drug Metab Pharmacokinet* 2016; **41**: 403-12. <https://doi.org/10.1007/s13318-015-0260-y>

246. Yang M, Wang AQ, Padilha EC*, et al.* Use of physiological based pharmacokinetic modeling for cross-species prediction of pharmacokinetic and tissue distribution profiles of a novel niclosamide prodrug. *Front Pharmacol* 2023; **14**: 1099425. <https://doi.org/10.3389/fphar.2023.1099425>

247. Yao X, Ye F, Zhang M*, et al.* In vitro antiviral activity and projection of optimized dosing design of hydroxychloroquine for the treatment of severe acute respiratory syndrome coronavirus 2 (SARS-CoV-2). *Clin Infect Dis* 2020; **71**: 732-9. <https://doi.org/10.1093/cid/ciaa237>

248. Zang X, Kagan L. Physiologically-based modeling and interspecies prediction of paclitaxel pharmacokinetics. *J Pharmacokinet Pharmacodyn* 2018; **45**: 577-92. <https://doi.org/10.1007/s10928-018-9586-9>

249. Zhang M, Yao X, Hou Z*, et al.* Development of a Physiologically Based Pharmacokinetic Model for Hydroxychloroquine and Its Application in Dose Optimization in Specific COVID-19 Patients. *Front Pharmacol* 2020; **11**: 585021. <https://doi.org/10.3389/fphar.2020.585021>

250. Zhu S, Zhang J, Lv Z*, et al.* Prediction of Tissue Exposures of Meropenem, Colistin, and Sulbactam in Pediatrics Using Physiologically Based Pharmacokinetic Modeling. *Clin Pharmacokinet* 2022; **61**: 1427-41. <https://doi.org/10.1007/s40262-022-01161-y>

251. Zurlinden TJ, Eppers GJ, Reisfeld B. Physiologically Based Pharmacokinetic Model of Rifapentine and 25-Desacetyl Rifapentine Disposition in Humans. *Antimicrob Agents Chemother* 2016; **60**: 4860-8. <https://doi.org/10.1128/aac.00031-16>

252. Valitalo PAJ, Griffioen K, Rizk ML*, et al.* Structure-Based Prediction of Anti-infective Drug Concentrations in the Human Lung Epithelial Lining Fluid. *Pharm Res* 2016; **33**: 856-67. <https://doi.org/10.1007/s11095-015-1832-x>

253. Aulin LBS, Valitalo PA, Rizk ML*, et al.* Validation of a Model Predicting Anti-infective Lung Penetration in the Epithelial Lining Fluid of Humans. *Pharm Res* 2018; **35**: 26. <https://doi.org/10.1007/s11095-017-2336-7>

# **Supplementary materials 3**: Preferred reporting items for systematic review and meta-analysis (PRISMA) checklists

# Table S6 PRISMA 2020 Abstracts Checklist

| **Section and Topic** | **Item #** | **Checklist item** | **Reported (Yes/No)** |
| --- | --- | --- | --- |
| **TITLE** | | |  |
| Title | 1 | Identify the report as a systematic review. | Yes |
| **BACKGROUND** | | |  |
| Objectives | 2 | Provide an explicit statement of the main objective(s) or question(s) the review addresses. | Yes |
| **METHODS** | | |  |
| Eligibility criteria | 3 | Specify the inclusion and exclusion criteria for the review. | No |
| Information sources | 4 | Specify the information sources (e.g. databases, registers) used to identify studies and the date when each was last searched. | Yes |
| Risk of bias | 5 | Specify the methods used to assess risk of bias in the included studies. | No |
| Synthesis of results | 6 | Specify the methods used to present and synthesise results. | Yes |
| **RESULTS** | | |  |
| Included studies | 7 | Give the total number of included studies and participants and summarise relevant characteristics of studies. | Yes |
| Synthesis of results | 8 | Present results for main outcomes, preferably indicating the number of included studies and participants for each. If meta-analysis was done, report the summary estimate and confidence/credible interval. If comparing groups, indicate the direction of the effect (i.e. which group is favoured). | Yes |
| **DISCUSSION** | | |  |
| Limitations of evidence | 9 | Provide a brief summary of the limitations of the evidence included in the review (e.g. study risk of bias, inconsistency and imprecision). | No, N.A. |
| Interpretation | 10 | Provide a general interpretation of the results and important implications. | Yes |
| **OTHER** | | |  |
| Funding | 11 | Specify the primary source of funding for the review. | Yes |
| Registration | 12 | Provide the register name and registration number. | No, N.A. |

# Table S7 PRISMA 2020 Checklist

| **Section and Topic** | **Item #** | **Checklist item** | **Where item is reported (section/chapter)** |
| --- | --- | --- | --- |
| **TITLE** | | |  |
| Title | 1 | Identify the report as a systematic review. | Title |
| **ABSTRACT** | | |  |
| Abstract | 2 | See the PRISMA 2020 for Abstracts checklist. | Abstract |
| **INTRODUCTION** | | |  |
| Rationale | 3 | Describe the rationale for the review in the context of existing knowledge. | 2 |
| Objectives | 4 | Provide an explicit statement of the objective(s) or question(s) the review addresses. | 2 |
| **METHODS** | | |  |
| Eligibility criteria | 5 | Specify the inclusion and exclusion criteria for the review and how studies were grouped for the syntheses. | 2, supplementary materials 1 |
| Information sources | 6 | Specify all databases, registers, websites, organisations, reference lists and other sources searched or consulted to identify studies. Specify the date when each source was last searched or consulted. | 2 |
| Search strategy | 7 | Present the full search strategies for all databases, registers and websites, including any filters and limits used. | Supplementary materials 1 |
| Selection process | 8 | Specify the methods used to decide whether a study met the inclusion criteria of the review, including how many reviewers screened each record and each report retrieved, whether they worked independently, and if applicable, details of automation tools used in the process. | 2 |
| Data collection process | 9 | Specify the methods used to collect data from reports, including how many reviewers collected data from each report, whether they worked independently, any processes for obtaining or confirming data from study investigators, and if applicable, details of automation tools used in the process. | 2 |
| Data items | 10a | List and define all outcomes for which data were sought. Specify whether all results that were compatible with each outcome domain in each study were sought (e.g. for all measures, time points, analyses), and if not, the methods used to decide which results to collect. | 2 |
|  | 10b | List and define all other variables for which data were sought (e.g. participant and intervention characteristics, funding sources). Describe any assumptions made about any missing or unclear information. | 2 |
| Study risk of bias assessment | 11 | Specify the methods used to assess risk of bias in the included studies, including details of the tool(s) used, how many reviewers assessed each study and whether they worked independently, and if applicable, details of automation tools used in the process. | N.A. |
| Effect measures | 12 | Specify for each outcome the effect measure(s) (e.g. risk ratio, mean difference) used in the synthesis or presentation of results. | N.A. |
| Synthesis methods | 13a | Describe the processes used to decide which studies were eligible for each synthesis (e.g. tabulating the study intervention characteristics and comparing against the planned groups for each synthesis (item #5)). | N.A. |
|  | 13b | Describe any methods required to prepare the data for presentation or synthesis, such as handling of missing summary statistics, or data conversions. | N.A. |
|  | 13c | Describe any methods used to tabulate or visually display results of individual studies and syntheses. | 2 |
|  | 13d | Describe any methods used to synthesize results and provide a rationale for the choice(s). If meta-analysis was performed, describe the model(s), method(s) to identify the presence and extent of statistical heterogeneity, and software package(s) used. | N.A. |
|  | 13e | Describe any methods used to explore possible causes of heterogeneity among study results (e.g. subgroup analysis, meta-regression). | N.A. |
|  | 13f | Describe any sensitivity analyses conducted to assess robustness of the synthesized results. | N.A. |
| Reporting bias assessment | 14 | Describe any methods used to assess risk of bias due to missing results in a synthesis (arising from reporting biases). | N.A. |
| Certainty assessment | 15 | Describe any methods used to assess certainty (or confidence) in the body of evidence for an outcome. | N.A. |
| **RESULTS** | | |  |
| Study selection | 16a | Describe the results of the search and selection process, from the number of records identified in the search to the number of studies included in the review, ideally using a flow diagram. | 3 |
|  | 16b | Cite studies that might appear to meet the inclusion criteria, but which were excluded, and explain why they were excluded. | 3 |
| Study characteristics | 17 | Cite each included study and present its characteristics. | Supplementary materials 2 |
| Risk of bias in studies | 18 | Present assessments of risk of bias for each included study. | N.A. |
| Results of individual studies | 19 | For all outcomes, present, for each study: (a) summary statistics for each group (where appropriate) and (b) an effect estimate and its precision (e.g. confidence/credible interval), ideally using structured tables or plots. | N.A. |
| Results of syntheses | 20a | For each synthesis, briefly summarise the characteristics and risk of bias among contributing studies. | N.A. |
|  | 20b | Present results of all statistical syntheses conducted. If meta-analysis was done, present for each the summary estimate and its precision (e.g. confidence/credible interval) and measures of statistical heterogeneity. If comparing groups, describe the direction of the effect. | N.A. |
|  | 20c | Present results of all investigations of possible causes of heterogeneity among study results. | N.A. |
|  | 20d | Present results of all sensitivity analyses conducted to assess the robustness of the synthesized results. | N.A. |
| Reporting biases | 21 | Present assessments of risk of bias due to missing results (arising from reporting biases) for each synthesis assessed. | N.A. |
| Certainty of evidence | 22 | Present assessments of certainty (or confidence) in the body of evidence for each outcome assessed. | N.A |
| **DISCUSSION** | | |  |
| Discussion | 23a | Provide a general interpretation of the results in the context of other evidence. | 7 |
|  | 23b | Discuss any limitations of the evidence included in the review. | N.A. |
|  | 23c | Discuss any limitations of the review processes used. | 7 |
|  | 23d | Discuss implications of the results for practice, policy, and future research. | 7 |
| **OTHER INFORMATION** | | |  |
| Registration and protocol | 24a | Provide registration information for the review, including register name and registration number, or state that the review was not registered. | Not registered |
|  | 24b | Indicate where the review protocol can be accessed, or state that a protocol was not prepared. | Not prepared |
|  | 24c | Describe and explain any amendments to information provided at registration or in the protocol. | N.A. |
| Support | 25 | Describe sources of financial or non-financial support for the review, and the role of the funders or sponsors in the review. | 7 |
| Competing interests | 26 | Declare any competing interests of review authors. | 7 |
| Availability of data, code and other materials | 27 | Report which of the following are publicly available and where they can be found: template data collection forms; data extracted from included studies; data used for all analyses; analytic code; any other materials used in the review. | 2, supplementary materials 1 & 2 |
